# Histologic and biochemical alterations predict pulmonary mechanical dysfunction in aging mice with chronic lung inflammation

**DOI:** 10.1371/journal.pcbi.1005570

**Published:** 2017-08-24

**Authors:** Christopher B. Massa, Angela M. Groves, Smita U. Jaggernauth, Debra L. Laskin, Andrew J. Gow

**Affiliations:** Department of Pharmacology and Toxicology, The Ernest Mario School of Pharmacy, Rutgers University, Piscataway, New Jersey, United States of America; Virginia Commonwealth University, UNITED STATES

## Abstract

Both aging and chronic inflammation produce complex structural and biochemical alterations to the lung known to impact work of breathing. Mice deficient in surfactant protein D (Sftpd) develop progressive age-related lung pathology characterized by tissue destruction/remodeling, accumulation of foamy macrophages and alteration in surfactant composition. This study proposes to relate changes in tissue structure seen in normal aging and in chronic inflammation to altered lung mechanics using a computational model. Alterations in lung function in aging and Sftpd -/- mice have been inferred from fitting simple mechanical models to respiratory impedance data (*Z*_*rs*_), however interpretation has been confounded by the simultaneous presence of multiple coexisting pathophysiologic processes. In contrast to the inverse modeling approach, this study uses simulation from experimental measurements to recapitulate how aging and inflammation alter *Z*_*rs*_. Histologic and mechanical measurements were made in C57BL6/J mice and congenic Sftpd-/- mice at 8, 27 and 80 weeks of age (n = 8/group). An anatomic computational model based on published airway morphometry was developed and *Z*_*rs*_ was simulated between 0.5 and 20 Hz. End expiratory pressure dependent changes in airway caliber and recruitment were estimated from mechanical measurements. Tissue elements were simulated using the constant phase model of viscoelasticity. Baseline elastance distribution was estimated in 8-week-old wild type mice, and stochastically varied for each condition based on experimentally measured alteration in elastic fiber composition, alveolar geometry and surfactant composition. Weighing reduction in model error against increasing model complexity allowed for identification of essential features underlying mechanical pathology and their contribution to *Z*_*rs*_. Using a maximum likelihood approach, alteration in lung recruitment and diminished elastic fiber density were shown predictive of mechanical alteration at airway opening, to a greater extent than overt acinar wall destruction. Model-predicted deficits in PEEP-dependent lung recruitment correlate with altered lung lining fluid composition independent of age or genotype.

## Author summary

Aging and chronic inflammation produce complex changes to the structure of the lung including accumulation of cells and debris, thinning and destruction of air sacs, altered airway size and increased tendency for airway collapse. As these structural changes are observed concurrently, their individual contributions to altered lung function cannot readily be determined by conventional measurement of lung function. Our study employs a novel approach to identifying the age progression of these effects in mice with and without chronic lung inflammation. Histologic changes in lung tissue were incorporated into a computational model of the mouse lung and used to simulate measured changes in lung function. By incorporating experimentally measured factors into the model in a stepwise fashion, the contribution of destructive and remodeling processes to alterations in lung function can be assessed. This modeling approach provides a framework for determining the significance of structural changes to the altered function observed in complex lung pathologies such as emphysema and chronic obstructive pulmonary disease. Such an approach could be utilized to assess mechanisms by which compounds alter lung function and the capacity of specific therapies to produce improvements in lung function at the organ level.

## Introduction

The lung has a stylized architecture that is crucial for the efficient exchange of gases between the vasculature and the airways. Organ level mechanical function and the distribution of ventilation during health and disease are dependent upon the complex interactions between heterogeneous airway and parenchymal elements. Alterations in histologic structure and mechanical function of the lung are established consequences of aging [1, 2]. With increasing age or advancing, decreases in tissue elastin content and destruction of alveolar septae contribute to a loss of parenchymal elasticity, with resulting increases in lung volume [2]. In addition, these changes may alter parenchymal tethering forces, thus reducing airway caliber, particularly during expiration. These processes occur gradually with age and, due to robustness in the complex architecture of the tissue, structural alteration may progress without any apparent impact on gas exchange or quality of life [3].

In the setting of chronic lung inflammation, structural and mechanical alterations must be interpreted with respect to changes that occur with normal aging [4–6]. Mice deficient in Surfactant protein-D (Sftpd) develop chronic lung inflammation characterized by the persistence of enlarged activated macrophages, destruction of acinar walls with airspace dilation and altered composition of the lung lining fluid [7, 8]. These changes are minor, but observable in young mice, with the phenotype becoming progressively more severe with age [9]. Earlier work has demonstrated that mechanical function in Sftpd deficient mice mimics that observed in wild-type C57BL6/J mice at 8 [10]and 27 weeks of age, but diverges at 80 weeks [9]. At 80 weeks, respiratory system elastance is elevated relative to C57BL6/J, a paradoxical observation given the emphysema-like histological change. Each of the aforementioned factors characterizing the complex pathology of Sftpd-/- mice is individually expected to contribute to respiratory system dysfunction, however the extent to which each contributes to the observed mechanical defect is unknown.

Respiratory impedance, as measured at airway opening is widely regarded as a sensitive indicator of pulmonary pathology in lung disease [4, 11–15]. Impedance measurements are typically analyzed using inverse modeling, where spectral data are fit to simple mechanical models with a small number of free parameters. Due to the complex and multiscale nature of the physical forces that influence lung mechanics, discrete biochemical and histological alterations that occur in pathology rarely relate directly to obvious changes in the impedance spectra or to changes in the model parameters. An alternative strategy for studying these concurrent processes is forward modeling [16–18], whereby a simulation of pathology is constructed based on perturbations to a control condition based on empiric data. In order to examine how specific pathologic observations may influence organ level function, the present study employs an anatomic computational model of the murine lung for forward simulation of pulmonary mechanics. These simulations utilize measured histological changes in acinar wall number and elastic fiber thickness as well as changes to lung recruitment in order to simulate respiratory impedance as a function of positive end expiratory pressure (PEEP). Incorporation of experimentally determined alterations into the model allows prediction of the magnitude of each effect when compared to experimental *Z*_*rs*_. In this way, the study is designed not to identify *the* mechanism by which pathology occurs, but to address the extent to which proposed mechanisms may contribute to altered mechanical function. Simulations were designed to test the hypothesis that destruction of alveolar septae and reduction in parenchymal elastic fiber thickness underlie the changes in lung resistance and elastance seen with age. Additionally, simulations were used to test the proposal that accelerated loss of tissue architecture, infiltration of the airspaces by cellular and crystalline material and loss of surfactant homeostasis secondary to chronic inflammation in Sftpd-/- mice are essential components of the complex mechanical phenotype seen in these mice.

## Materials and methods

### Animal care and use

Experiments were performed in accordance to Rutgers University IACUC approved protocols conforming to the NIH guidelines for the care and use of laboratory animals. Male wild-type C57BL6/J and congenic Sftpd -/- mice were bred at Rutgers University under the care of Laboratory Animal Services. All mice were housed in micro-isolation cages under sterile conditions with food and water provided ad libitum. Mice were examined at 8, 27 and 80 weeks of age. General anesthesia was induced via single intraperitoneal injection of ketamine and xylazine. At 6 minutes post injection withdrawal from footpad pinch was used to determine depth of anesthesia. Surgical tracheosteomy was performed under aseptic technique. Following lung function measurement, animals were euthanized while on the ventilator by exsanguination via aortic incision with concurrent en-bloc perfusion of the lungs via instillation of heparinized saline through the right ventricle.

### Measurement of lung mechanics

Lung mechanical function was assessed as previously described [9]. Briefly, anesthetized mice (N = 8 per group) were ventilated using the Flexivent Small Animal Ventilator (Scireq, Montreal QC) via tracheostomy, at 120 breaths/minute and a tidal volume of 10mL/kg body weight. Mechanics were assessed at 5 positive end expiratory pressures (PEEPs) of 0, 1, 3, 6 and 9 cm H_2_O. Following equilibration at each PEEP forced oscillation measurements and quasi-static pressure volume loops were generated in triplicate, with each measurement perturbation separated by 15 s of normal tidal ventilation.

Forced oscillation measurements were made by transducing pressure during a flow-controlled broad-band waveform composed of 17 sinusoidal waveforms with mutually prime non-integer frequencies between 0.5 and 20 Hz, lasting 8 s. Respiratory system impedance, *Z*_*rs*_, was calculated as the ratio of the Fourier transformed pressure to flow signals as a function of frequency. From this, respiratory resistance, reactance and elastance were determined from the real and imaginary portions of *Z*_*rs*_. Constant phase parameters were estimated off-line using a Nelder-Mead simplex method in Matlab (Mathworks, Natick, MA).

Sub-maximal quasi-static pressure-volume (PV) loops were generated as previously described [19]. Briefly, the lung underwent stepwise inflation to a peak pressure 10 cm H_2_O above PEEP in 8 equal-magnitude pressure-regulated steps over 16 s. PV hysteresis was calculated as the area between the inspiratory and expiratory limbs of the loop. Elastance was calculated as the ΔV/ΔP across the entire PV loop as well as for each incremental pressure step.

### Bronchoalveolar lavage (BAL) collection and analysis

BAL was performed using 4 x 1mL washes with saline as previously described [9]. Cells were removed by centrifugation at 300x g for 10 min. Supernatant was frozen at -80°C. Samples were defrosted and centrifuged at 20,000x g for 1 hr to separate BAL into large and small aggregate fractions. Large aggregate phospholipid content was determined by the method of Dyer and Bligh with each sample run in triplicate [20]. Relative SP-B content in the large aggregate fraction was determined by western blot with each lane loaded for constant phospholipid content. Gels were run under reducing conditions and imaged using horseradish peroxidase catalyzed chemiluminesence (ECL prime, GE Life Sciences, Pittsburgh, PA) and quantified by Bio-rad gel imager. Phospholipid content and SP-B intensity were normalized to control (8-week-old C57BL6/J mice), analyzed by 2-way ANOVA on genotype and age followed by Welch’s post-hoc test (p<0.05 for significance) and reported as mean ± standard error.

### Histology

Following BAL collection, the lung was inflated to TLC using 25 cm H_2_O with 2% Paraformaldehyde, 3% sucrose. The trachea was tied to maintain inflation, incubated overnight and then transferred to 70% ethanol. Tissues were paraffin embedded and sectioned at 5 μm thickness. Tissue sections were imaged at 100x and 400x magnification using the Olympus VS-120 microscope. Hematoxylin and eosin staining was used for assessment of radial alveolar counts (RAC) and measurement of obstruction of the parenchymal space by inflammatory cells and/or crystalline and hyaline debris. RAC was determined by counting the number of alveolar tissue septae traversed by a chord drawn perpendicular from a terminal bronchiole to the lung edge [9, 21]. The RAC is thus not a measure of acinar volume, but is proportional to the number of intact tissue septa and will decrease as acinar walls undergo destruction, but will not change in the case of airspace dilatation. All terminal bronchioles within a 10 x view from a pleural surface or a connective tissue septum were used for counting. Counts were made by two blinded observers on 3–4 mice per condition, with 15–20 counts made per slide to generate a robust distribution. The fraction of tissue obstructed was measured as the percentage of the parenchymal airspaces filled with either enlarged immune cells or acellular debris in each section.

Tissue collagen and elastic fiber content was examined using a Modified Verhoeff-VanGeisson stain technique (Sigma-Aldrich St. Louis, MO). For each mouse at least 10 high powered fields were analyzed, with the thickness of each parenchymal elastic fiber measured using ImageJ. Fiber thickness was measured in triplicate and averaged, with most slides having 15–20 parenchymal fibers per field; fibers lining airways and vasculature rather than airspace were excluded from analysis.

### Forward modeling

**Fig 1:**
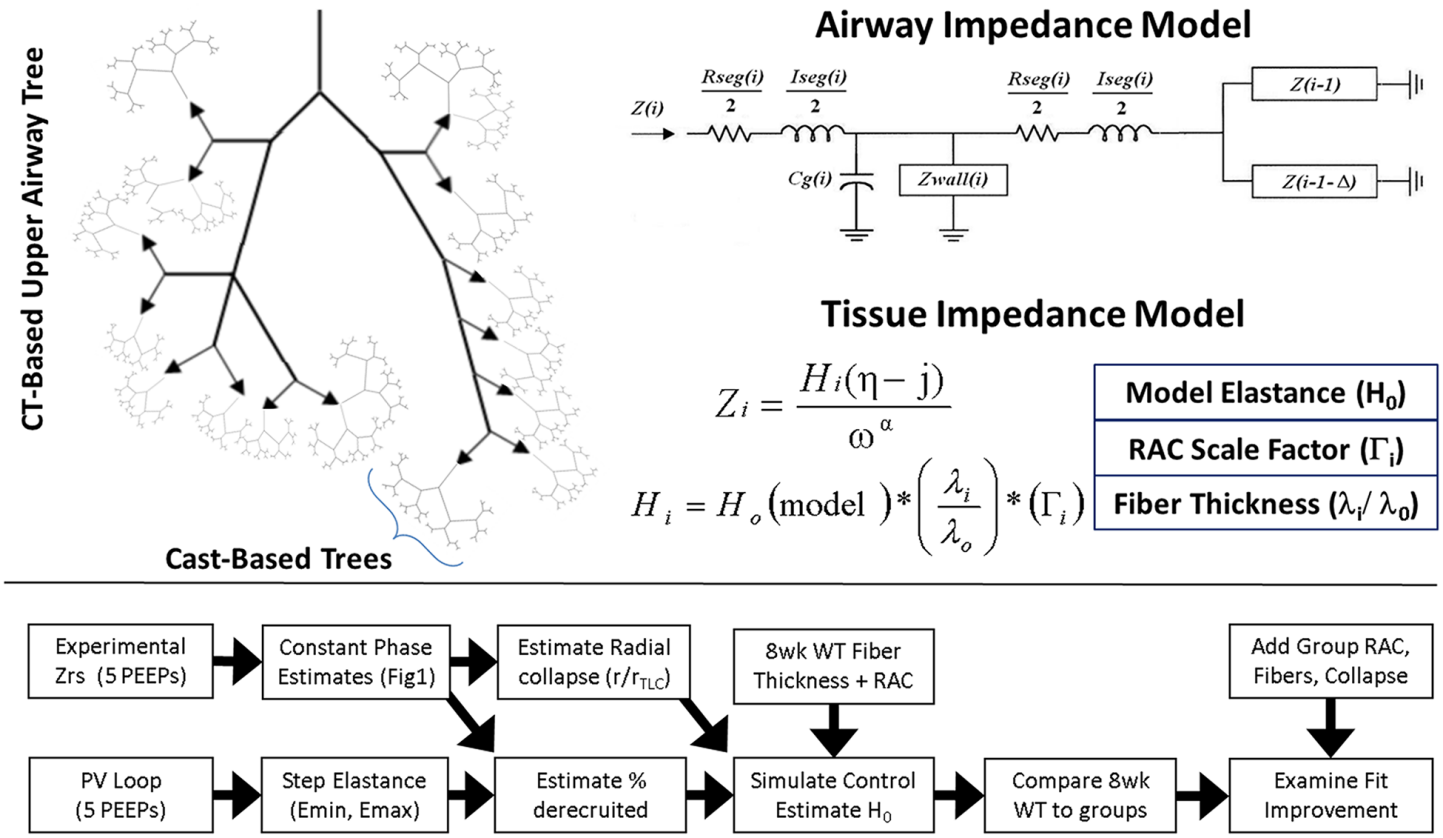
Model architecture and simulation strategy. Simulation of respiratory impedance employs an anatomic description hybridized from CT and plaster cast data from the literature (A). Tree impedance was calculated using airway lengths and radii and a recursive algorithm to traverse the tree (B). Tissue properties incorporated an estimated model base elastance, radial alveolar count, and fiber thickness (C). Simulation strategy employs mechanical data to construct a model of the 8week WT mouse and determines reduction in error by increasing model complexity (D).

An anatomic model of the airway tree was developed by hybridizing CT-based structural information from the C57Bl6/J mouse upper airway tree with measurements made from silicone casts of the micromus lung. For the upper portion of the tree, airways greater than 1 mm in diameter were assigned a mean radius and length based on micro CT imaging [22]. Below this threshold radius, the subtending airway network was modeled based on the recursive branching model structure of the micromus lung [23]. At the transition from CT-based to cast based-model the generation of the two daughter branches was determined based on matching the length and radii of the parent airway to the nearest order within the cast model. Branching pattern beneath this juncture was dichotomous, with each order *n*, being subtended by orders *n-1* and *n-1-Δ*_*n*_. The branching nature of the upper airway tree is illustrated in Fig 1. The volume of each airway was calculated as *V*_*seg*_ = *πr*^2^*l*. Total volume of the modeled tree was compared to the estimated airway volume at TLC using a recursive algorithm to traverse the entire structure beginning at the trachea: $V_{n}=V_{seg\left(n\right)}+V_{n-1}+V_{n-1-\Delta_{n}}$. The simulation was run repeatedly to determine a mean and standard deviation of the total volume of the airway tree at TLC and found to agree within 2% of the volume estimate for the mouse lung at 25 cmH_2_O (0.152 vs 0.155 mL) [23].

Impedance modeling was performed as previously described and are described briefly, with detailed equations given in the included supplement (S1 Appendix). Mechanical properties for each airway were based on the length, *l*, and radius *r*, of each segment, *i*, which are determined stochastically based on airway order. Impedance of each airway segment contained a resistance given by Poiseuille’s law, an inertial component from gas acceleration, as well as parallel contributions from the distension of airway walls and gas compression [16]. Airway wall distension and gas compression may contribute significantly to lung impedance during pathology and were simulated as viscoelastic based on airway geometry [24, 25]. Parallel impedances were modeled as acting at the airway midpoint, bisecting the longitudinal impedance into components acting proximal and distal to compression/distension processes. The impedance into a given branch of the tree can be determined by combining the impedances of the subtending branches in parallel, adding this in series to the distal longitudinal impedance [17]. This sum is combined in parallel with gas compression and wall distension impedances, and then the total is added in series to the proximal longitudinal impedance [16].

A recursive algorithm was employed to generate the branching nature of the tree. As the cast-based tree branches dichotomously, the impedance was readily calculated using standard approaches for bifurcating networks [16] at each transition point where the anatomical definition changes from CT based to plaster-cast based. As no recursive relationship exists for the CT-based upper airway tree, each cast-based network was simulated individually and added as the downstream impedances to ends of the CT based tree. The impedance network of the upper airway tree was then constructed by appropriate combination of downstream impedances in parallel and series until reaching the trachea, at which point the impedance of the lungs, *Z*_*L*_, is fully computed.

Tissue contributions to *Z*_*L*_ were simulated by adding viscoelastic constant phase tissue elements to the terminal branches of the tree structure described above as previously described [26]. Impedance of the chest wall is considered negligible in mice, and was omitted [27]. All simulations were performed at the 17 frequencies below 20Hz to match experimental measurements.

### Simulation strategy

A hierarchical modeling approach was undertaken to evaluate the impact of alveolar wall destruction, elastic fiber thinning, and airway derecruitment on lung resistance and elastance. (Fig 1). First, the effects of aging and loss of Sftpd on airway caliber were examined. Airway radii were scaled by constant value minimizing the error in airway resistance between the model tree and experimental data. Using the scaled airway tree, the control condition (8-week old C57Bl6/J recruited to PEEP 6 cmH_2_O) was simulated with tissue elastance distributions generated from the radial alveolar count and elastic fiber thickness measurements. The contribution of each of the above factors on error between simulated and measured *Z*_*rs*_ spectra was determined by replacing control tissue properties with those from the appropriate age-genotype distribution. In order to determine the role of PEEP-dependent acinar recruitment, probabilistic collapse of terminal tissue elements was incorporated into the model based on estimates of minimum tissue elastance from the Pressure-Volume curve [28].

### Estimating changes to airway caliber with PEEP

As the geometry of each airway generation was determined from measurements made at TLC, it was necessary to scale the airway radii in order to match the measured resistance spectra for each experimental condition as a function of PEEP. Simulation of *Z*_*rs*_ was performed at each PEEP for all conditions by setting the tissue mechanical properties equal to estimated values of H and η from the constant phase model, and performing a one dimensional optimization on a constant multiplier applied to all radii. This scalar factor, *r/r*_*0*_, constrained between 0 and 1 was estimated by bisection method to reduce the sum of squared residuals between model and experimental *Z*_*rs*_ data. As radii in the tree are stochastic, simulations were repeated 100 times per condition and reported as a mean and standard deviation. All subsequent simulations were run with *r/r*_*0*_ set as the mean of the radial scale factor distribution at each PEEP.

### Modeling tissue properties based on histological criteria

Heterogeneity in tissue mechanics is modeled using extracellular matrix composition or variation in alveolar wall number within the acinus. Simulations were performed using measurements of these factors from tissue histology. As these properties naturally vary within the tissue, measurements were treated as a discrete random variable and pooled across all samples within a condition to generate a probability distribution function. A total of 8 models for generating elastance distributions are proposed– homogenous lung, plus three ways of incorporating RAC, each with or without an elastic fiber contribution.

In order to generate the tissue elastance distribution a baseline elastance, *H*_*0*_, unique to each of the proposed models was estimated. This was accomplished by minimizing the error between the simulated anatomic model impedance and the measured *Z*_*rs*_ spectra from maximally recruited control mice (8-week-old C57Bl6/J mice at PEEP of 6 cm H_2_O) by least squares optimization on the single variable *H*_*0*_. The value of the *H*_*0*_ parameter can be considered the intrinsic stiffness of a reference alveolar unit upon which the distribution of model stiffness is based. Each terminal tissue unit is assigned an individual elastance, *H*_*i*_, value drawn from a distribution influenced by 4 possible factors: *H*_*0*_ (*model*), elastic fiber thickness, λ, radial alveolar count, Γ_i_, and PEEP-dependent probability of derecruitment, *p*_*collapse*_

$$H_{i}=\left\{\begin{array}{cc} H_{0}\left(model\right)\times \left(\lambda \right)\times \left(\Gamma_{\text{i}}\right) & Rand_{i}\geq p_{collapse}\\ Inf & Rand_{i}<p_{collapse} \end{array}\right\}$$ (1)

Incorporation of elastic fiber thickness into the model began with pooling the thickness measurements across subjects for each condition. A thickness measurement, λ_*i*_, was drawn at random for each of the terminal elements in the tree. Tissue elastance was scaled based on the assumption that fiber thickness linearly related to the stiffness, so that the elastance of a given unit was scaled based on λ_*i*_ so that *H*_*i*_ ∝ (*λ*_*i*_/*λ*_0_) where λ_0_ is the average thickness of the control distribution.

$$H_{i}=\left\{\begin{array}{cc} H_{0}\left(model\right)\times \left(\frac{\lambda_{i}}{\lambda_{0}}\right)\times \left(\Gamma_{\text{i}}\right) & Rand_{i}\geq p_{collapse}\\ Inf & Rand_{i}<p_{collapse} \end{array}\right\}$$ (2)

Acinar destruction with alveolar wall loss was incorporated into the simulation using the radial alveolar count. For each terminal tissue unit an individual radial alveolar count measurement (*RAC*_*i*_) was randomly selected from the experimental RAC distribution and value for Γ_i_ determined by comparing it to a value RAC_crit_. All sampling was done with replacement. Three methods of relating radial alveolar count to tissue elastance distributions were examined and are detailed in the supplemental material (Appendix). Briefly, the model variants include: 1) comparing *RAC*_*i*_ to a fixed threshold (*RAC*_*crit*_ = 12), if [*RAC*_*i*_ > *RAC*_*crit*_ Γ_i_ = 1; *RAC*_*i*_ < *RAC*_*crit*_ Γ_i_ = 0.5]. 2): comparing *RAC*_*i*_ to a random *RAC*_*crit*_ drawn from the control distribution, if [*RAC*_*i*_ > *RAC*_*crit*_ Γ_i_ = 1; *RAC*_*i*_ < *RAC*_*crit*_ Γ_i_ = 0.5]. 3) comparing *RAC*_*i*_ to a fixed threshold (*RAC*_*crit*_ = 12), and scaling elastance hyperbolically—if [*RAC*_*i*_ > *RAC*_*crit*_ Γ_i_ = 1; *RAC*_*i*_ < *RAC*_*crit*_ Γ_i_ = (*RAC*_*i*_ / *RAC*_*crit*_)].

The probability of a terminal element being nonventilated, *p*_*collapse*_, was modeled as a PEEP dependent phenomenon based on elastance changes within the PV loops, estimates of the constant phase parameter H and the % tissue opacification from tissue histology. The quasi-static nature of the PV loop permits computation of effective elastance at each step in the maneuver. Elastance of the fully recruited lung was estimated as the nadir of the step elastance curve during progressive recruitment, *E*_*min*_, as previously published [19, 28] and is detailed in the supplemental methods. This elastance minimum was incorporated into a simple model of 1000 parallel elastance units allowed to be recruited or derecruited such that total lung elastance is hyperbolically related to the number of open pathways. The fraction of recruitable lung which is open was thus estimated as $\;f_{open}=\frac{H_{tissue}}{{\;}_{N_{total}\;xH_{CP}\left(PEEP\right)}}$ where the ratio *H*_*tissue*_/*N*_*total*_ is the elastance of the fully recruited lung—*E*_*min*_ above—and *H*_*CP*_(*PEEP*) is the constant phase elastance value at a given level of PEEP.

Each condition was run as a monte-carlo simulation with 100 replicates; the mean and variation of all simulated spectra and estimated parameters quantified. Coefficient of variation of impedance spectra for all simulated experimental conditions was < 10% under every model of tissue mechanical behavior presented.

### Model error determination and comparison

For each simulated model spectrum goodness of fit to the experimental data was given by the total error, calculated as:

$$\phi_{M}=\sum_{K=1}^{17}\left[{\left(R_{L}\left(f_{K}\right)-R_{M}\left(f_{K}\right)\right)}^{2}+{\left(X_{L}\left(f_{K}\right)-X_{M}\left(f_{K}\right)\right)}^{2}\right]$$ (3)

where *R*_*L*_ and *X*_*L*_ are the resistance and reactance spectra of the experimental data and *R*_*L*_ and *X*_*L*_ are the simulated spectra under model *M*. In order to compare the ability of each modeled factor on their contribution to the recapitulation of mechanical phenotype, each model goodness-of-fit must be compared between simulations. As these simulations are not fit to the data using optimization, conventional approaches to comparing models are not appropriate. Although such statistical hypothesis testing and information criteria based approaches cannot be employed, model likelihood ratios can be computed as

$$L_{M}=-2log\left(\sum {\left[R_{L}\left(f_{K}\right)-R_{M}\left(f_{K}\right)\right]}^{2}+\sum {\left[X_{L}\left(f_{K}\right)-X_{M}\left(f_{K}\right)\right]}^{2}\right)+2log\left(\sum {\left[R_{L}\left(f_{K}\right)-R_{0}\left(f_{K}\right)\right]}^{2}+\sum {\left[X_{L}\left(f_{K}\right)-X_{0}\left(f_{K}\right)\right]}^{2}\right)$$

where *R*_*0*_ and *X*_*0*_ are the simulated resistance and reactance spectra of the null model (8wk WT distributions). Though this strategy gives direct quantitative comparison, it is limited in that increasing model complexity is not explicitly penalized.

## Results

### Pulmonary mechanics

**Fig 2:**
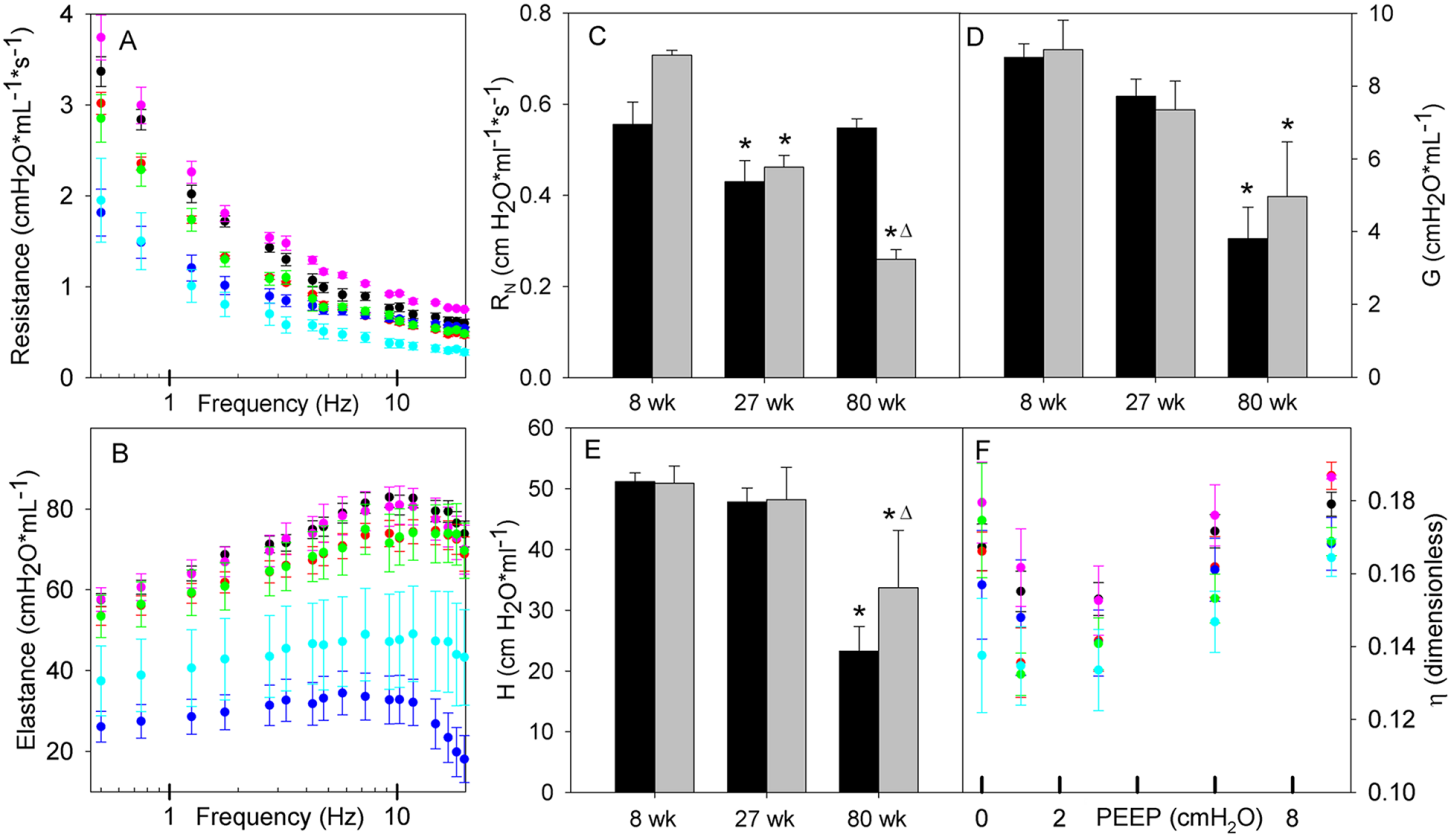
Respiratory impedance spectra with constant phase parameters by age and genotype. Panels A and B show lung resistance and elastance spectra at PEEP of 6 cmH2O as a function of oscillatory frequency. Data are reported as mean +/- standard error with WT mice at 8 (black), 27 (red) and 80 weeks (blue), Sftpd-/- at 8 (pink), 27 (green) and 80 weeks (cyan). Spectra were not found significantly different with PEEP, however a significant age effect and age-genotype interaction were detected. Estimated constant phase model parameters (Rn, G, H, η) are shown in panels C-F, reported as mean+/- standard error. As Rn, G and H are invariant with PEEP, values are shown at PEEP 6 only, with solid bars indicating WT and gray bars for Sftpd-/-. PEEP responsiveness is shown for η. All parameters were analyzed using 3way ANOVA on genotype, age and PEEP. For all parameters, a significant age effect is noted, with a genotype-age interaction present in Rn, G and H. Genotype effect alone is significant for Rn and H. Significance: (* p<0.05 vs. 8 week control in same genotype. Δ: p<0.05 vs. WT at same age).

Respiratory mechanical properties were assessed by forced oscillation and by quasi-static pressure volume loop. These data are complementary examining both static and dynamic respiratory properties as a function of PEEP ranging from 0 to 9 cm H_2_O. Respiratory resistance and elastance spectra are presented for a PEEP of 6 cm H_2_O (Fig 2, panels A and B), chosen as a PEEP that balances maximal recruitment with minimal overdistension across the conditions. *Z*_*rs*_ spectra were mostly PEEP independent, with the inertial effects on high frequency E_L_ spectra being the most affected by increasing end expiratory pressure (S1 Fig). At the PEEP of 6 cm H_2_O, E_L_ spectra at 8 and 27 weeks are indistinguishable from each other irrespective of genotype (Similar results were observed at a PEEP of 3 cm H_2_O). At 80 weeks of age C57BL6/J mice have a significantly lower E_L_ spectrum than Sftpd(-/-) mice. There is a progressive fall in the R_L_ spectra with increasing age independent of genotype (Fig 2A). At 80 weeks of age there is a divergence of the high frequency spectra in the Sftpd(-/-) mice, which was observable at all levels of PEEP.

Constant phase model parameters are useful in anatomically compartmentalizing these changes, placing them in a physiologic context and providing a quantification of lung properties for the basis of modeling the pathology. Parameters were examined using 3-way ANOVA on age, genotype and PEEP, followed by Dunnett’s post hoc test (p<0.05) All PEEP trends in constant phase parameter values are presented in S2 Fig, with salient features shown in Fig 2, panels C-F. The frequency invariant airway resistance, R_N_, demonstrates significant effects in genotype, age and their interaction. At 8 weeks, R_N_ is significantly elevated with the loss of Sftpd (Fig 2C) and increases as a function of PEEP. At 27 weeks, R_N_ is lower than at 8 weeks but there is no longer a dependence on genotype. At 80 weeks, the values for R_N_ have significantly diverged, with C57BL6/J mice demonstrating a resistance close to that of 8-week-old mice and Sftpd(-/-) mice being dramatically reduced. Tissue resistance, G, demonstrates age and age-genotype interactions, with a trend similar to that seen in elastance (Fig 2D). Estimated elastance, *H*, becomes significantly lower with age and demonstrates dependence on genotype, but not PEEP. There is a significant interaction between Age and genotype, showing that loss of Sftpd reduces the magnitude by which H falls at 80 weeks compared to C57BL6/J mice (Fig 2E). Tissue hysteresivity, η defined as the ratio of G to H, is significantly effected by age and PEEP, with increasing age tending to decrease η, and PEEP effect being non-monotonic, with a minimum value at either PEEP of 1 or 3 cm H_2_O (Fig 2F).

**Fig 3:**
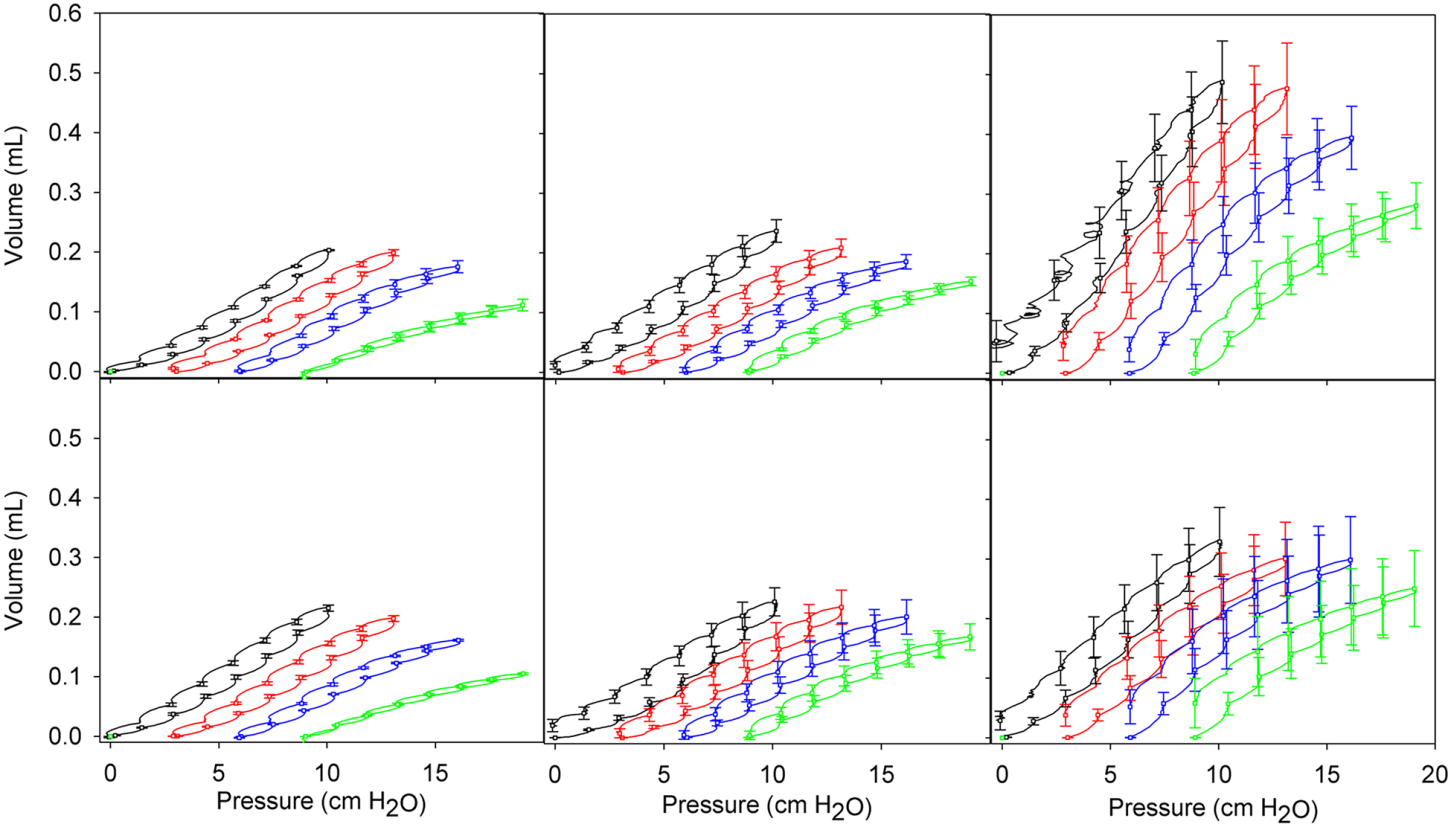
Quasi-static pressure-volume relationships for each group as a function of PEEP. Panels A-C are 8, 27 and 80 weeks WT mice; Panels D-F are 8, 27 and 80 week Sftpd KO mice. Curves are colored for each PEEP: 0 (black), 3 (red), 6 (blue) and 9 (green) cm H2O. Data are presented as mean +/- standard error.

Pressure volume curves were analyzed to detect changes in lung recruitment throughout the process of quasi-static inflation (Fig 3). In all mice, increasing PEEP reduces the peak volume reached for the PV loop, as expected as the lung approaches TLC. Peak lung volume during the PV maneuver is unchanged at 27 weeks of age, but is significantly increased in 80 week old mice. With loss of Sftpd age-related volume increase is significant but is reduced relative to that in C57BL6/J mice. For 8 and 27 week old mice the PV area monotonically decreases with increasing PEEP. This relationship is maintained in 80 week Sftpd(-/-) mice, however, in C57Bl6/J mice there is non-monotonic dependency (Table 1). This indicates an increased pressure dependence for recruitment with age, and shows that the Sftpd(-/-) lung is harder to recruit by increasing PEEP.

**Table 1:** PV loop area. Area between the inflation and deflation limb of PV curves, as calculated by integration are presented as mean +/- standard error. Units are cmH_2_O*mL^-3^. Data were analyzed using 3 way ANOVA, followed by Dunnet’s post-hoc test (p < .05 for significance).

PEEP (cmH_2_O)		0	1	3	6	9
WT	8	0.31 ± 0.020	0.30 ± 0.021	0.34 ± 0.022	0.26 ± 0.024	0.01 ± 0.030
	27	0.44 ± 0.028 *	0.38 ± 0.027 *	0.38 ± 0.026	0.33 ± 0.028	0.23 ± 0.034 *
	80	0.57 ± 0.084 *	0.70 ± 0.084 *	0.91 ± 0.083 *	0.86 ± 0.074 *	0.68 ± 0.056 *
Sftpd -/-	8	0.35 ± 0.029 Δ	0.32 ± 0.028 Δ	0.35 ± 0.029	0.25 ± 0.031	-0.01 ± 0.04
	27	0.48 ± 0.024 *	0.41 ± 0.024 *	0.35 ± 0.026	0.33 ± 0.023	0.24 ± 0.020
	80	0.58 ± 0.043 *	0.59 ± 0.031 *	0.6 ± 0.016 *Δ	0.79 ± 0.006 *Δ	0.94 ± 0.021 *Δ

* indicates significance relative to 8 week within genotype,

^Δ^ represents significant change vs WT at of the same age.

Elastance over the entire PV loop gradually increases with PEEP in both 8-week-old C57Bl6/J and Sftpd(-/-) mice. However, at a PEEP of 9 cm H_2_O there is a marked increase in stiffness, as is expected with strain stiffening behavior (Table 2A). At 27 weeks loop elastance closely mimics that seen in 8-week-old mice, however, the strain stiffening behavior is blunted in both genotypes. There is a considerable loss of elastance over the PV loop at 80 weeks of age in both genotypes. However, there are significant differences between the genotypes. 80-week-old Sftpd(-/-) mice have elastance measurements higher than those of old C57Bl6/J mice, however, there is minimal PEEP dependence with no significant strain stiffening at a PEEP of 9 cm H_2_O. These observations are consistent with a loss of fiber integrity in both genotypes, but with increased recruitment in the Sftpd(-/-) allowing for maintenance of lung elastance at low PEEP despite the surfactant abnormalities seen in these mice. A similar trend is observed in the minimum step elastance values reached during PV maneuvers (Table 2B).

**Table 2:** Estimated elastance parameters from quasi-static PV loops. Derived elastance parameters estimated from PV curves at each PEEP. Estimates of whole-loop elastance (A) and minimum values for the step elastance (B) are presented as mean +/- standard error. Units are cmH_2_O/mL. Parameters were analyzed using 3 way ANOVA, followed by Dunnet’s post-hoc test (p < .05 for significance).

PEEP (cmH_2_O)		0	1	3	6	9
**A**						
WT	8	48.3 ± 1.51	49.5 ± 1.43	50.0 ± 1.45	56.9 ± 2.14	86.2 ± 4.57
	27	44.0 ± 2.70	48.6 ± 2.17	50.4 ± 3.28	56.0 ± 3.35	68.1 ± 2.94 *
	80	22.3 ± 2.71*	22.4 ± 3.02*	23.0 ± 3.28*	27.2 ± 3.28*	38.1 ± 4.63 *
Sftpd -/-	8	45.3 ± 1.64	47.8 ± 2.29	49.0 ± 2.43	59.1 ± 3.21	91.0 ± 4.71
	27	47.3 ± 4.84	49.0 ± 5.42	50.3 ± 6.10	55.1 ± 7.14	65.1 ± 8.38 *
	80	33.2 ± 5.65 *Δ	35.0 ± 6.11 *Δ	37.5 ± 7.42 *Δ	39.23 ± 8.69 *Δ	47.3 ± 10.82 *
**B**						
WT	8	36.2 ± 0.99	37.8 ± 1.00	40.3 ± 1.19	46.1 ± 1.54	67.4 ± 4.43
	27	32.6 ± 1.80	36.2 ± 2.60	40.6 ± 2.86	45.4 ± 2.90	54.7 ± 4.27 *
	80	14.7 ± 4.37 *	18.4 ± 3.11 *	20.0 ± 3.10 *	20.9 ± 3.72 *	28.3 ± 5.98 *
Sftpd -/-	8	35.4 ± 1.51	37.1 ± 1.62	40.0 ± 1.98	47.0 ± 2.70	68.8 ± 5.49
	27	34.0 ± 4.72	33.6 ± 5.47	40.6 ± 4.88	43.9 ± 5.41	52.4 ± 8.44 *
	80	27.3 ± 5.46 *Δ	29.9 ± 6.61 Δ	32.6 ± 8.10 Δ	32.0 ± 10.99 *Δ	35.2 ± 14.02

* indicates significance relative to 8 weeks within genotype,

^Δ^ represents significant change vs. WT at of the same age.

### Histopathology

**Fig 4:**
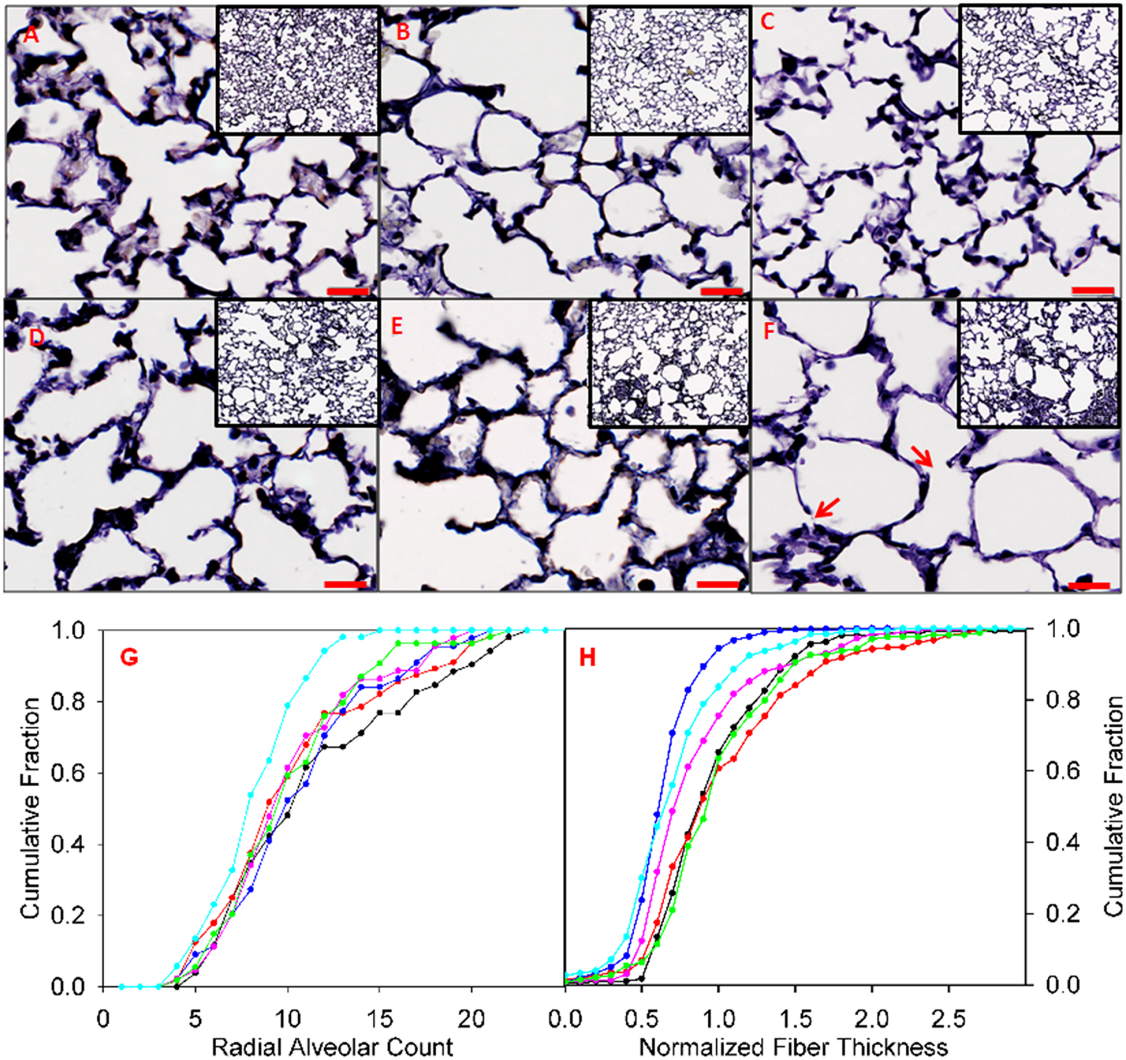
Lung tissue histology with quantification of radial alveolar count and normalized elastin fiber thickness. Images A-F: Voerhoff’s stained lung tissue is presented at 400x in large image (bar = 20 um), with 100 x. inset. Cumulative frequency distributions of radial alveolar counts (G) and elastin fiber thicknesses (H) were obtained by pooling measurements across mice within each group. WT mice at 8 (black), 27 (red) and 80 week (blue), Sftpd-/- at 8 (pink), 27 (green) and 80 week (cyan). Red arrows indicate apparent breaks in the alveolar wall.

As a means to assess alterations in lung structure, tissue sections stained with Verhoeff’s stain were examined by light microscopy following full inflation with paraformaldehyde and are shown at 100x and 400x magnification (Fig 4A–4F). At low magnification (inset), age related airspace enlargement is evident, with some apparent loss of septae, particularly in the subpleural acini. These changes are exacerbated by the loss of Sftpd, with more pronounced evidence of septal destruction at higher magnification. Quantification of septal loss using radial alveolar counts (RAC) was expressed as a cumulative probability distribution for each condition, where a leftward shift in the distribution reflects a loss of septae (Fig 4G). In C57Bl6/J mice, RAC is reduced to an equivalent extent in both 27 and 80-week-old mice. In 8-week-old Sftpd(-/-) mice there is a similar leftward shift in RAC distribution to that seen in 27 and 80-week-old C57Bl6/J mice. The loss of septae that occurs within Sftpd(-/-) mice is progressive, with the greatest loss of acinar walls being observed in 80-week-old mice. Thinning of the elastic fibers within the parenchyma is also evident with age (Fig 4H), accompanied by a decrease in the fraction of walls staining for elastin fibers. Within C57Bl6/J mice there is no change in fiber thickness at 27 weeks of age, but at 80 weeks there is a dramatic reduction. Relative to C57BL6/J, increased fiber thickness is observed in 8-week-old Sftpd(-/-) mice. However, this difference is not observed at 27 weeks of age. No significant obstruction of parenchymal space by hyaline, cellular or crystalline material was observed in the C57BL6/J mouse with age, however opacification of the airspaces was progressive with age in the Sftpd(-/-) mouse (8week = 3.4 ± 1.7%, 27 week = 6.8 ± 2.3%, 80 week = 13.8 ± 4.2%,).

### Model initialization

Initial estimation of model parameters is required in order to prepare the model for simulation of specific experimental data. From Fig 1 one can see that our modeling strategy requires three parameters r/r_0_ (a radial scaling factor as a fraction of radius at TLC), a measure of lung recruitability, and a baseline estimate of H_0_ for the 8-week-old C57Bl6/J mouse. These parameters can be combined with our experimental measurements of fiber thickness and RAC to generate a simulation (Fig 1). A r/r_0_ was estimated in order to map the TLC airway tree to the appropriate geometry at each PEEP. As parenchymal destruction may alter tethering forces, a value for r/r_0_ was estimated at each PEEP for each experimental condition (Table 3). Airway radial scale factors follow the trend in PEEP, age and genotype predicted by R_N_ estimates. Notably, r/r_0_ are significantly divergent at 80 weeks of age, falling in the C57Bl6/J mouse but increasing significantly in the Sftpd(-/-). Estimation of the fraction of recruitable lung, which is ventilated at a given PEEP was estimated from pressure-volume data and constant phase estimates (Table 4). The approach undertaken matches a-priori predictions with PEEP levels from 1 to 6 cm H_2_O, but fits less well at extreme levels of PEEP (0 and 9 cm H_2_O), where tissue hysteresivity changes due to extensive collapse or strain stiffening. In the physiological PEEP range, the fraction of recruited lung increases linearly with PEEP, though the extent of collapse is age and genotype dependent. At 8 weeks, the Sftpd(-/-) has greater recruitment than WT at low PEEP, but peaks at the same % opening with recruitment. By 27 weeks, the fraction of recruitable lung was lower in the Sftpd(-/-) at PEEP of 1 cm H_2_O, but overlapped at all higher levels of PEEP. The recruited fraction continues to increase up to a PEEP of 9 cm H_2_O, in contrast to 8-week-old mice. At 80 weeks of age, C57Bl6/J mice follow a similar recruitment trend to that seen at 27 weeks, with evidence of maximal recruitment and strain stiffening at 9 cm H_2_O. In 80-week-old Sftpd(-/-) mice the fraction of recruitable lung was significantly elevated over all other conditions, and is near 100% at 9 cm H_2_O.

**Table 3:** Estimates of r/r0 from model initialization. Model optimization produced estimates of a normalized radius r/r0. Data are reported as mean +/- standard deviation following 100 independent optimization routines per condition.

PEEP (cmH_2_O)		0	1	3	6	9
WT	8 week	0.66 ± 0.025	0.64 ± 0.015	0.60 ± 0.015	0.53 ± 0.007	0.54 ± 0.008
	27 week	0.53 ± 0.020	0.55 ± 0.007	0.56 ± 0.007	0.57 ± 0.009	0.61 ± 0.011
	80 week	0.47 ± 0.009	0.49 ± 0.008	0.50 ± 0.010	0.1 ± 0.020	0.51 ± 0.020
Sftpd -/-	8 week	0.61 ± 0.015	0.59 ± 0.012	0.54 ± 0.010	0.50 ± 0.009	0.51 ± 0.010
	27 week	0.51 ± 0.010	0.52 ± 0.010	0.55 ± 0.005	0.56 ± 0.007	0.58 ± 0.010
	80 week	0.62 ± 0.010	0.62 ± 0.010	0.62 ± 0.010	0.66 ± 0.015	0.69 ± 0.015

**Table 4:** Estimated fraction of non-obstructed terminal units recruited. Estimated lung recruitment reported as a fraction of potentially recruitable terminal elements as a function of PEEP. Values are extrapolated from changes in the PV loop assuming fixed acinar elastance, H0. Data are reported as mean +/- standard deviation following 100 independent optimization routines per condition.

PEEP (cmH_2_O)		0	1	3	6	9
WT	8 week	0.73 ± 0.015	0.71 ± 0.010	0.74 ± 0.009	0.79 ± 0.015	0.71 ± 0.013
	27 week	0.91 ± 0.022	0.75 ± 0.010	0.78 ± 0.010	0.85 ± 0.015	0.88 ± 0.015
	80 week	0.70 ± 0.020	0.73 ± 0.010	0.77 ± 0.010	0.86 ± 0.015	0.82 ± 0.015
Sftpd -/-	8 week	0.77 ± 0.013	0.73 ± 0.011	0.77 ± 0.010	0.79 ± 0.011	0.71 ± 0.018
	27 week	0.87 ± 0.010	0.71 ± 0.010	0.78 ± 0.013	0.84 ± 0.015	0.89 ± 0.020
	80 week	0.86 ± 0.018	0.82 ± 0.015	0.84 ± 0.018	0.95 ± 0.018	1.00 ± 0.015

For each model, the value of H_0_ was estimated at PEEP 6 in the 8-week-old C57Bl6/J mouse, and remained fixed for simulation of all other conditions. All models were fit on the single parameter H_0_ by reduction of the sum of squared residuals between model and *Z*_*rs*_ data. Error and H_0_ values are reported as the mean and standard deviation of 100 optimizations per model (Table 5). All 8 models tested converged reliably and produced parameter estimates in the physiologic range. Residual error was nearly equivalent for 6 of the 8 models proposed. Notably, the model with the poorest fit when optimized is the sole model to assume homogenous parenchymal mechanics.

**Table 5:** Estimated values of H0 for each proposed model. Estimated values of H0 for each proposed model. Units are cmH_2_O/mL. Model optimization generated estimates of H0 from 8week WT data, with small residual error. Data are reported as mean +/- standard deviation following 100 independent optimization routines per model.

RAC Model		0	1	2	3
No Fibers	Error	0.13 ± 0.011	0.03 ± 0.004	0.03 ± 0.003	0.03 ± 0.003
	H0	28.3 ± 7.89	69.9 ± 2.23	73.1 ± 3.45	56.9 ± 2.73
With Fibers	Error	0.04 ± 0.008	0.09 ± 0.025	0.04 ± .009	0.05 ± 0.019
	H0	51.1 ± 2.54	72.9 ± 4.76	83.5 ± 2.61	67.2 ± 2.98

### Simulation of experimental data

**Fig 5:**
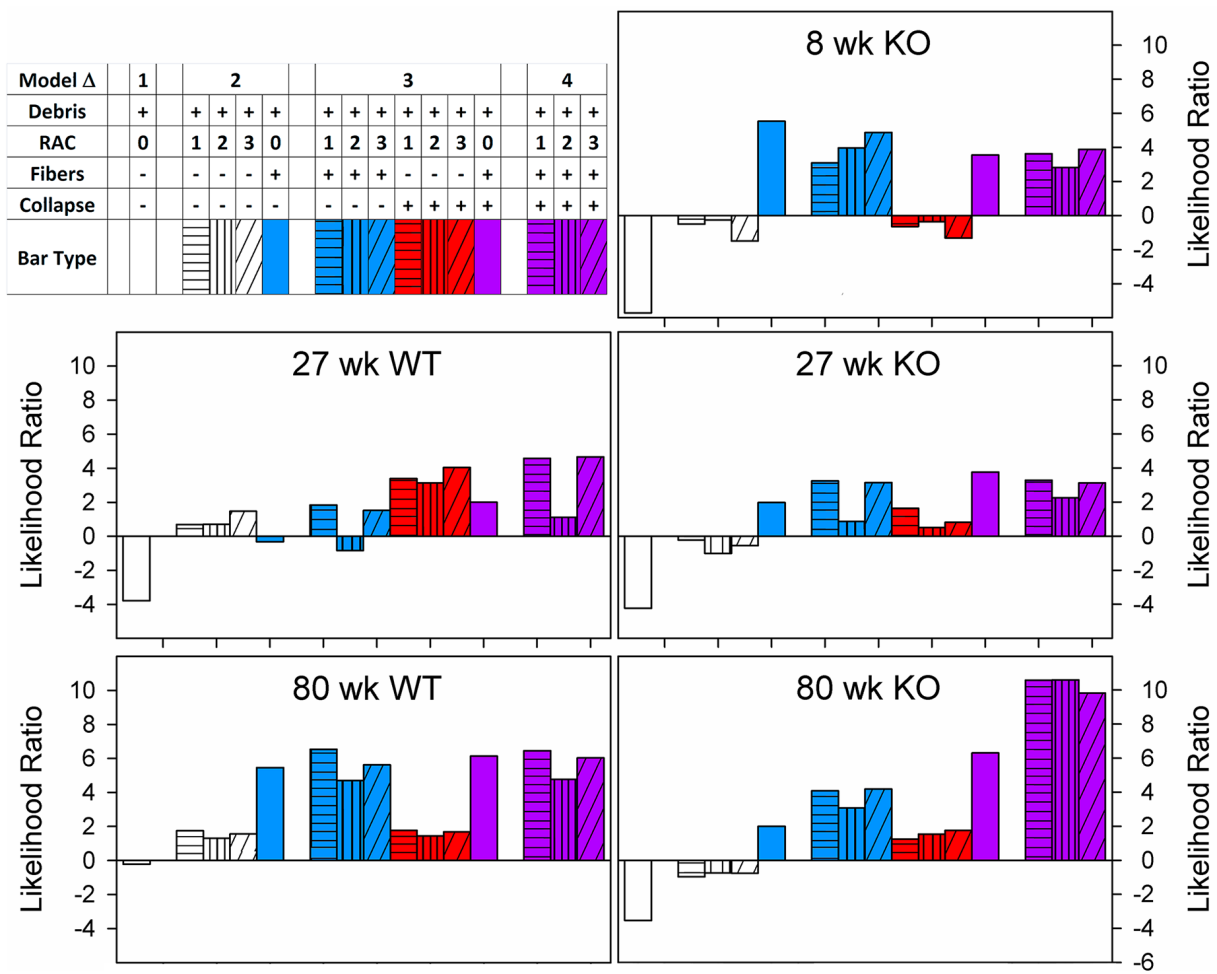
Model likelihood is shown for each genotype and age as a function of model complexity. All models normalized to 8 week WT mouse. Models are shade-coded by RAC model, and color coded by inclusion of fiber thickness (blue), stochastic derecruitment (red) or both (purple). Models are separated into groups by the number of perturbations used in that simulation using empty space, so that model complexity can be appreciated. Models increased in complexity from left to right. For most simulations, fiber thickness is the single perturbation that provides the greatest increase in model likelihood. Derecruitment provides only modest improvement, except in 27 week WT and 80 week KO. In the 80 week WT, substantial improvement in model fit is observed with addition of all factors into the model.

The error between each condition and the 8-week C57Bl6/J mouse was calculated at a PEEP of 6 cm H_2_O for the H_0_ model. There was no significant difference in goodness of fit for the 8-week-old Sftpd(-/-) (ε = .9844 times the control error). At 27 weeks, both the C57Bl6/J and Sftpd(-/-) mice resulted in an increase in error by 61.2 and 62.6% respectively. At 80 weeks, this error increased dramatically, 5.2 fold for the C57Bl6/J, and 3.8 fold for the Sftpd(-/-). With the exception of 27-week-old C57Bl6/J mice, incorporation of elastic fibers was the single most effective perturbation to the model in terms of reduction of error. RAC alone was successful in reducing the error in 27-week C57Bl6/J mice and 27 and 80-week Sftpd(-/-) mice. PEEP dependent derecruitment potentiated the reduction in model error produced by RAC based models in 27-week-old C57Bl6/J and Sftpd(-/-) mice and 80-week-old Sftpd(-/-) mice. When all factors were incorporated the model error was significantly improved in the 80-week Sftpd(-/-) mouse, otherwise only modest benefit was observed for the incorporation of additional complexity. The relative contributions of each change are further quantified using the likelihood ratios (Fig 5) in the paragraph below and PEEP dependent error (S3 Supporting Material).

As the purpose of these simulations was to discern the relative significance of each factor, we used likelihood ratios without hypothesis testing to compare model fitness (Fig 5). In C57Bl6/J mice at 27 weeks RAC incorporation, but not fiber thickness results in increased model likelihood. Incorporation of differential collapse is critical for improving model likelihood, increasing the log-likelihood ratio of each model roughly 2.5 fold. The 80-week C57Bl6/J mice are poorly characterized by RAC based models and depend critically on elastic fiber thickness distributions.

In all Sftpd(-/-) mice elastic fiber distributions drive an improvement in model fit. While the goodness of fit in the 8-week Sftpd(-/-) mouse is not affected by incorporation of lung collapse, the benefit of fiber thickness is diminished by the addition of RAC. In the 27-week-old Sftpd(-/-) elastic fibers are the most important single factor for improvement of model fit, with PEEP dependent collapse but not RAC potentiating the improvement in model likelihood. At 80 weeks, both RAC and fiber thickness improve model likelihood, however their combination results in a dramatic reduction in error that synergizes with PEEP dependent collapse. In these mice, a model incorporating all factors has greater than a 10 fold log-likelihood vs control, corroborating the importance of derecruitment, fiber thickness and alveolar wall destruction in this pathology. Simulated spectra from each maximum likelihood model is shown in the supplemental data (S5 Fig)

Although model comparison was performed at PEEP of 6, observed *Z*_*rs*_ spectra demonstrate some PEEP dependence (S1 Fig). To determine the extent by which these changes result from altered recruitment, additional simulations were run for each model at every PEEP with and without appropriate changes in the recruited fraction of terminal units, as estimated from the experimental P-V loops. As a measure of PEEP dependence, the variance of the model error was calculated across the 5 levels of PEEP under both simulated recruitment conditions (S3 Fig). In each experimental condition and for every model the simple addition of estimated recruitment eliminates virtually all of the PEEP dependence in the error between data and the model. The extent to which derecruitment explains the PEEP variance was expressed as the ratio of the variances measured across levels of PEEP with PV-based derecruitment to those without. Error variance with PEEP was effectively reduced to less than 10% of original model variance in 30 out of 48 simulations, with the 80-week C57Bl6/J mouse demonstrating the least PEEP responsiveness (S4 Fig). No particular model was demonstrably less responsive to PEEP across experimental conditions, though models incorporating only fiber thickness showed generally less error reduction with derecruitment.

### Potential role for surfactant dysfunction in enhanced collapse

**Fig 6:**
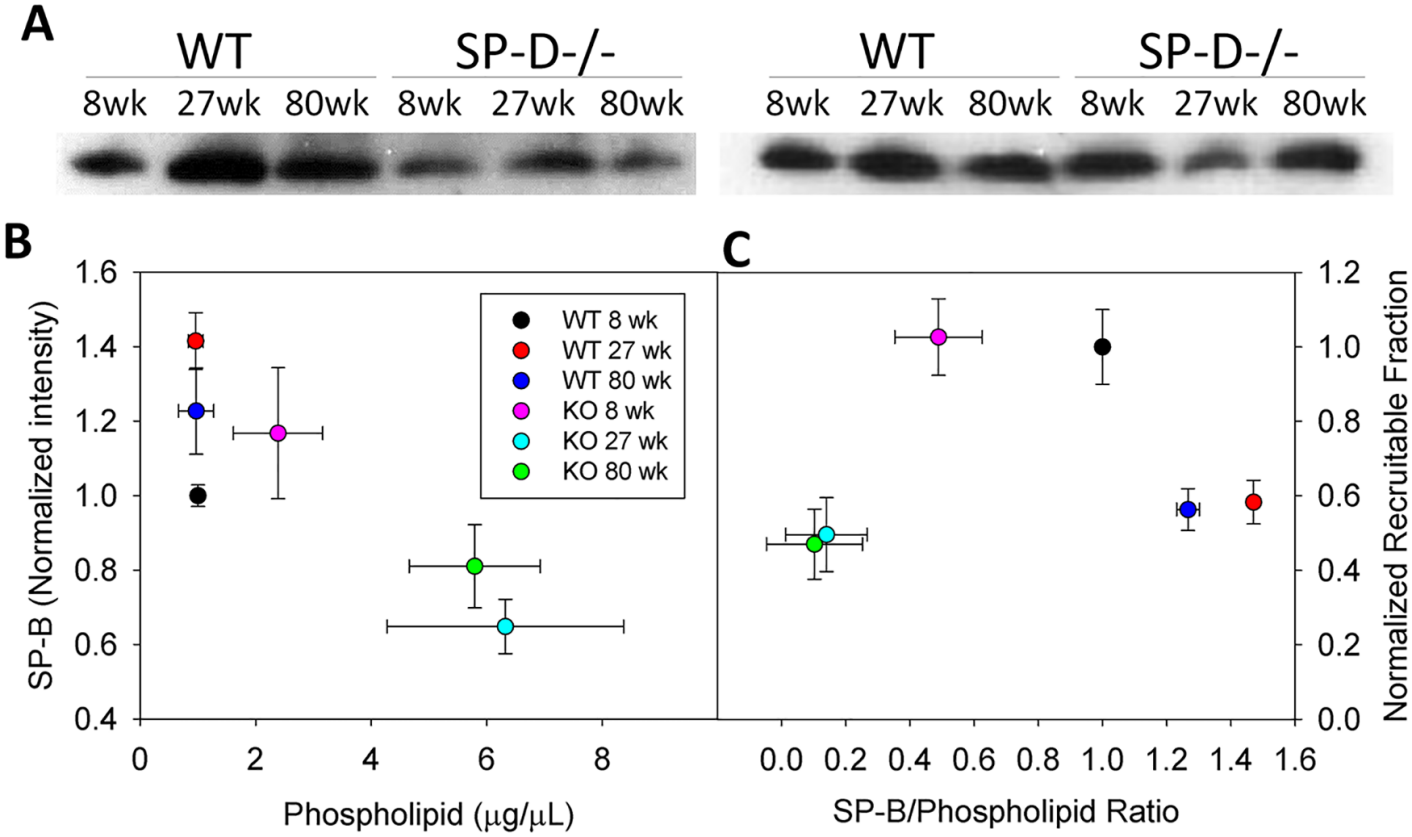
Analysis of BAL large aggregate fraction SP-B and phospholipid. Western blot for SP-B in BAL Large aggregate fraction is shown in A. Two blots shown are from different mice. SP-B blots were quantified by densitometry and plotted as a function of BAL phospholipid content (B). The predicted change in recruited fraction with increasing PEEP was normalized to WT at 8 week and plotted versus the change in SP-B/Phospholipid ratio (C). All data reported as mean +/- standard error.

Maintenance of an appropriate surfactant protein to phospholipid ratio is essential for trafficking of surface-active material between the hypophase and air-liquid interface. Hence phospholipid and protein content is near optimized for maintaining small airway patency and excursions either above or below the physiologic level are deleterious. Dramatic accumulation of phospholipid occurs within the lungs of Sftpd(-/-) mice. We examined this change as a potential mechanism for the observed increased propensity for collapse. Mechanistic data relating BAL composition directly to surface-active function and recruitment are not available for incorporation into the model; however, we examined the relationship between alteration of these quantities and estimated recruitment (Fig 6). These data show a relationship between altered SP-B to phospholipid ratio and reduced recruitable fraction of terminal lung units.

## Discussion

Alterations to parenchymal architecture have been proposed to underlie changes to pulmonary function that occur with aging [1, 2] and chronic inflammation [29]. Studies attempting to relate complex heterogeneous changes in lung architecture to altered mechanical function are scarce and largely have not employed simulation. The goal of the present studies were to examine the effects of aging and chronic inflammation upon both lung architecture and function, and to determine, via a modeling approach whether there is a mechanistic link. We have used C57Bl6/J mice as a healthy control and Sftpd(-/-), on the same background, as a model of the chronically inflamed lung. We examined these mice at three ages: 8, 27, and 80 weeks. Using a variety of histological techniques, acinar septal destruction, reduction in elastin fiber content, and accumulation of inflammatory cells and debris were quantified as a function of both aging and loss of Sftpd. These measurements were made in conjunction with assessment of lung mechanical function by both forced oscillation and quasi-static pressure volume curves at a range of PEEPs. Both lung function and architecture reflect emphysematous tissue destruction in the aging C57Bl6/J mouse. The extensive tissue destruction that is observed in the aging Sftpd(-/-) mouse, is most evident when comparing functional measurements at 80-weeks of age, where tissue elastance and resistance increase relative to the C57Bl6/J while R_N_ falls. These observations are consistent with a loss of parenchymal integrity and a consequent increase in airway diameter. A forward computational model, simulating the impedance spectra, was used to demonstrate the significance of the various tissue alterations as well as PEEP-dependent alterations to recruitment in producing the mechanical phenotype observed. Using this approach it appears that fiber thickness is a major component of the functional alterations seen both with aging and chronic inflammation, while lung collapse plays a significant role in the 80-week-old Sftpd(-/-) mice. This study demonstrates the value of combining structural and functional measurements and provides a valuable tool for the pre-clinical assessment of lung anti-inflammatory agents.

Both aging and loss of Sftpd result in profound alterations in lung tissue architecture. As predicted with aging, mice undergo a reduction in RAC and both elastin fiber number and thickness. These changes are potentiated by the loss of Sftpd; in particular there is a significant reduction in RAC at all ages in Sftpd(-/-) when compared to C57Bl6/J. Although stereology represents the gold standard for alterations in lung architecture [30], it is important to note that these observations are in agreement with previous work that has shown a significant loss of alveolar number and a consequent increase in mean alveolar size in 12-week-old Sftpd(-/-) mice by detailed stereological analysis [31]. Similarly, computational simulation has allowed partitioning of NOS2 dependent and independent contributions to lung mechanical dysfunction using stereological measurements in 29 week old WT, Sftpd(-/-) and dual Sftpd(-/-)/NOS2(-/-) mice [28]. In this study elastin fiber characteristics and RAC were considered as though they were truly independent quantities, however, loss of elastin fiber within a wall may predispose it to strain-mediated rupture, producing a right shift in fiber thickness distribution with a fall in RAC. Furthermore, as fiber thickness is measured from scanned images of dye-stained slides, there is a lower limit of resolution below which a fiber may exist, but be too thin for detection or accurate measurement. A secondary limitation occurs whereby the Sftpd (-/-) mouse undergoes deposition of cellular, hyaline and crystalline debris, which was modeled exclusively as a cause for stationary obstruction. In fact, these regions of collapse may act to increase the stiffness of adjacent acinar structures by restricting their expansion. Such second order effects of collapse could be modeled using in-vivo imaging with co-registration to map areas of inflammation to specific anatomic regions of the tree [32].

Within this study we have used a hybridized CT-plaster cast reconstruction of the airway anatomy at TLC. The combination of this airway anatomy with *Z*_*rs*_ spectra collected at varying levels of PEEP allowed for the estimation of an airway scaling factor r/r_0_ (Table 3) that provides a measure of the changes in airway caliber with age and genotype. When we combine these measures with the recruitable lung fraction (Table 4), as estimated from the PEEP-dependent changes in the quasi-static PV loops (Fig 3), we were able to make the following conclusions. With aging in the C57Bl6/J mouse there is a loss of airway tree volume as the parenchymal volume increases. The loss of airway caliber may explain why R_N_ remains relatively unchanged with age (Fig 2), when one may have predicted a fall with increasing tissue destruction. In contrast within Sftpd(-/-) mice, at a given value of PEEP, parenchymal recruitment increases with age (Table 4) as does r/r_0_. This increasing airway caliber no doubt contributes to the age-dependent fall in R_N_ seen in this genotype (Fig 2).

Elastin fiber thickness incorporation is the single factor, which reduces simulation error most substantially and consistently across age and loss of Sftpd (Fig 4). Prior studies of young Sftpd(-/-) mice have shown that additional loss of inducible nitric oxide synthase ameliorates the tissue mechanical change and airspace dilation, supporting a role for immune-mediated elastin degradation [31]. Other experimental models which produce emphysematous change—including exposure to cigarette smoke, elastase instillation or alpha-1 antitrypsin deficiency—have mechanistically supported a loss of elastin underlying increases in lung volume [33, 34]. Overt septal destruction observed in these models is often considered causative in altering lung mechanics, yet altered fiber properties may contribute more significantly to the phenotype, particularly before a threshold for tissue destruction is crossed. While incorporation of septal destruction using the RAC improved model fit, particularly in aging mice; the improvement seen was no better than if differential recruitment had been added.

The quasi-static PV loops show a decrease in the maximal volume of each loop as PEEP increases, consistent with ventilation above a higher recruited lung volume. As mice age the total lung volume increases, as is clearly observable at 80 weeks of age. This increase is blunted with Sftpd(-/-), congruent with the extensive airspace obstruction observed on histology. That the PV loop area falls with increasing PEEP at both 8 and 27 weeks of age indicates that pressure support is not necessary for maximal recruitment over the measurement interval. Indeed, at a PEEP of 9 cm H_2_O there is a considerable increase in whole-loop elastance consistent with a strain stiffening effect in the lung tissue. However, at 80 weeks of age there is a need for pressure support to maximally recruit the Sftpd(-/-) lung; as shown by the lack of a PEEP-dependent fall in PV loop height and by a reduced strain stiffening effect. In light of these observations, it is significant that only within the 80-week-old Sftpd(-/-) mouse is there a need to include a lung collapse component to maximize model fit. These observations confirm the value in our multi-component forward modeling of lung function, as multiple parameters are internally consistent.

The probity of our approach, and the possibility of using other techniques to improve simulation authenticity, are provided by examining the changes in SP-B content. While the addition of differential derecruitment from the P-V loop showed minimal effect on model error at PEEP of 6 cm H_2_O, a dramatic reduction in the error variance across PEEPs was observed (S1 Table). This is despite constant phase parameter estimates demonstrating little PEEP dependence (S1 Fig). Such addition of a single measured mechanical metric of recruitment to account for such great proportion of PEEP dependent change in model performance is additionally consistent with a role for airway collapse as the mechanism for differential PEEP dependence in *Z*_*rs*_ spectra. The benefit of adding derecruitment appears associated with the extent with which BAL composition is disrupted, with alterations in SP-B content predicting both an increase in required model complexity and reduction in error variance with PEEP.

### Model/Study limitations

This study uses structural and mechanical measurements to simulate age and inflammation related lung dysfunction in a murine model of emphysema. There is disagreement in the literature as to collagen and elastin content, organization and crosslinking in human aging and chronic lung disease [35–39]. Such disparities may become even greater when considering differences between rodents and humans [40]. The C57BL6/J mouse at 80 weeks has previously been reported to have reduced elastin without changes in collagen content [1]. Histology in this study is consistent with these data, however collagen was not specifically quantified. As fibrillar collagens principally contribute to mechanics at higher lung volumes, their impact on low amplitude forced oscillation measurements may be minimal, however they are expected to contribute to apparent strain stiffening of the PV curves. In considering the nonlinear dependence of tissue rheology on specific collagen isoform content, fiber size distribution and the extent of intra-microfibrillar and inter-microfibrillar crosslinking recapitulating such phenomena would require extensive characterization of the tissue ultrastructure. Stereologic quantification, particularly in conjunction with electron microscopy, though technically demanding, would be a uniquely robust strategy for assessing these changes in tissue structure. Were such detailed tissue investigation to be performed, particularly in an experimental model of fibrosis, these alterations could be incorporated into the described computational strategy with ease.

The inability to mechanistically link surfactant composition to organ level lung mechanics is another limitation of this work. Though disruption of PEEP-dependent changes in PV loop are correlated with surfactant alteration, this is presented solely as an empirical observation. Ideally, the SP-B and phospholipid data would be incorporated into a model that predicts the pressure dependence of recruitment and derecruitment. To date, no physical or empirical relationship has been proposed to relate these measurements with either organ level or ex-vivo surfactant function. An alternative approach to such modeling could incorporate direct measurements of surfactant function using ex-vivo surfactometry, such as the Langmuir-Blodgett trough. Such a study that quantitatively relates protein and lipid content to both surface active function and in vivo mechanics would represent a major advance in the study of lung mechanics. This, too would further increase this potential applicability of this study’s strategy to acute lung injury and the neonatal respiratory distress syndrome.

The multi-faceted approach presented here which included detailed histological analysis in conjunction with functional assessment allows for accurate forward modeling of lung function. This is a novel approach that allows for direct examination of how changes in lung structure affect respiratory function and the relative importance of these changes. Such integrated measurement and modeling approaches may in fact be required to relate heterogeneous structural and functional measurements across multiple length scales as a result of the inherent complexity in biological systems. We propose that the use of this modeling approach may allow for assessment of the mechanisms involved in the loss of lung function as a consequence of inflammatory disease.

## Supporting information

S1 Appendix Supplemental methods. (PDF)

S1 Fig Experimental *Z*_*rs*_ spectra. (PDF)

S2 Fig Estimated constant phase parameters. (PDF)

S3 Fig Residual model errors with and without differential recruitment. (PDF)

S4 Fig Tissue elastance distribution histograms. (PDF)

S5 Fig Representative RL and EL spectra. (PDF)

S1 Table PEEP dependent error variance. (PDF)
