# Supplementary material for: Histologic and biochemical alterations predict pulmonary mechanical dysfunction in aging mice with chronic lung inflammation
Source: PLoS Comput Biol. 2017 Aug 24;13(8):e1005570. doi: 10.1371/journal.pcbi.1005570 (PMC5570219; doi:10.1371/journal.pcbi.1005570)
Supplement: S1 Appendix — (PDF) [file pcbi.1005570.s001.pdf]

Supplemental Material 1: Detailed model development

Airway tree impedance calculations

For each segment, *i*, segment properties are principally determined by a Newtonian airway resistance ($R_{seg}(i)$), a linear inertia term based on the energy expended to accelerate a mass of moving gas ($I_{seg}(i)$), and

$$R_{seg}(i) = \frac{8\mu l_i}{\pi {r_i}^4}$$

$$I_{seg}(i) = \frac{\rho l_i}{\pi {r_i}^2}$$

where μ and ρ represent the viscosity and density of air respectively. As the pressure and flow signals produced experimentally are relatively small in magnitude, contributions to impedance associated with turbulent flow and bifurcations have been neglected. The sum of these two factors was considered the segment longitudinal impedance, $Z_{seg}(i)$.

$$Z_{seg}(i) = R_{seg}(i) + j\omega * I_{seg}(i)$$

In order to model wall distension, airway wall thickness, *h*, was modeled as a function of radius using the empiric relationship derived from the small airways in a dog (Horsfield et.al., 1982):

$$h(r) = 0.0856 * r + 0.0417. \quad R^2 = 0.972.$$

Each wall segment was assumed to have resistive, inertial and compliant components of its impedance based on estimated lung tissue kinematic viscosity, ν_L, density, ρ_L, and young's modulus, Y_L, respectively (Habib et.al., 1993):

$$Z_{wall}(i) = R_{wall}(i) + j\left(\omega * I_{wall}(i) - \frac{C_{wall}(i)}{\omega}\right)$$

where *j* is the unit imaginary number, ω is the angular frequency in radians/second and

$$R_{wall}(i) = \frac{h\nu_L}{2\pi l_i {r_i}^3}$$

$$I_{wall}(i) = \frac{2h_i\rho_L}{\pi l_i r_i}$$

and

$$C_{wall}(i) = \frac{\pi l_i r_i}{2h_i Y_L}$$

Energy storage in adiabatic gas compression ($Cg_{seg}(i)$) was proportional to segment volume and inversely related to atmospheric pressure:

$$Cg_{seg}(i) = \frac{Vseg_i}{Patm} = \frac{\pi {r_i}^2 l_i}{Patm}$$

producing an impedance contribution of

$$Z_{g(i)} = -j\left(\frac{Cg_i(i)}{\omega}\right)$$

Wall impedance and gas compression are incorporated into the airway model as parallel processes to the movement of gas through the tube, acting at the midpoint of the tube. This divides the segment impedance into 2 halves, one proximal (in series) and one distal (in parallel) to the wall distension and gas compression:

$$Z_{in} = \frac{Z_{seg(i)}}{2} + \left(Z_{wall(i)} \parallel Z_{g(i)} \parallel \frac{Z_{seg(i)}}{2}\right)$$

In order to simulate impedance at airway opening an algorithm is called recursively beginning at the trachea. This algorithm combines the impedences of the two daughter branches in parallel, then adds this in series to the airway segment impedance calculation. Because of the parallel contribution of gas compression and wall distension this can be calculated as:

$$Z_i = \frac{Z_{seg(i)}}{2} + \left(Z_{wall(i)} \parallel Z_{g(i)} \parallel \left(\frac{Z_{seg(i)}}{2} + \left(Z_{i-1} \parallel Z_{i-1-\Delta_i}\right)\right)\right)$$

As the impedance looking into each generation depend on the impedance further down the tree, calling the above function results in two function calls to the subtending daughter branches; this proceeds until a terminal acinar structure is reached (generation 1). Acinar mechanics are modeled as having a viscoelastic component obeying constant phase mechanical properties in parallel with a gas compression compliance calculated as ambient pressure at sea level divided by approximate acinar volume (total lung volume – calculated volume of the whole airway tree)

$$Z_{acn} = \left(\frac{H(j-\eta)}{\omega^{\alpha}}\right) \parallel \left(\frac{-P_{Atm}}{j\omega * \mathrm{V}_{\mathrm{Acn}}}\right)$$

where η is the ratio of elastic to dissipative forces of the tissue (hysteresivity), and $\alpha = 2/\pi * \arctan(1/\eta)$.

Relating acinar destruction to tissue mechanics through RAC

Three models by which acinar wall loss could alter tissue mechanics (Γ_i) were utilized in these simulations. Alveolar wall loss (Γ_i) was modeled using the radial alveolar count by drawing an individual count (RAC_i) from the sampled RAC distribution and used to calculate a tissue elastance value. Three methods of relating radial alveolar count to tissue elastance distributions were examined:

1) Using a fixed cutoff for RAC, reduce the elastance of the tissue unit by half based on RAC_i. This cutoff, RAC_{crit}, was chosen to be the point where the control RAC distribution undergoes a change in slope ($RAC_{crit} = 12$).

$$\Gamma_i = \begin{Bmatrix} 1 & RAC_i \geq RAC_{crit} \\ 0.5 & RAC_i < RAC_{crit} \end{Bmatrix}$$

2) For each terminal tssue element random values drawn from both the control and simulated condition RAC distribution are compared to determine the tissue element stiffness. The elastance value of a tissue unit will be reduced by half if the representative draw from the control, RAC_{crit}, is greater than that from the simulation condition, RAC_i. This allows the resulting elastance distribution to be determined by the heterogeneity of the sampled condition relative to that of the control mice.

$$\Gamma_i = \begin{Bmatrix} 1 & RAC_i \geq RAC_{crit} \\ 0.5 & RAC_i < RAC_{crit} \end{Bmatrix}$$

3) Using a fixed cutof**f** for RAC, ($RAC_{crit} = 12$) hyperbolically scale tissue elastance based on distance between RAC_i and a reference unit, RAC_{crit}, assuming that structural units can only be lost, not gained.

$$\Gamma_i = \begin{Bmatrix} 1 & RAC_i \geq RAC_{crit} \\ \dfrac{RAC_i}{RAC_{crit}} & RAC_i < RAC_{crit} \end{Bmatrix}$$

In addition to the above 3 models, two models were proposed which relate the inverse of RAC to an acinar chord length from which a normalized acinar volume could be computed. These volume-based models were found to simulate unreliably, with significant variability in parameter distributions and poor convergence properties when estimating H0. For this reason, these models were not included in the results. It is conceivable – though speculative - that those models failed due to a lack of thresholding effect, which has been observed in continuum mechanical simulations of tissue rheology.

Using a simple model of 1000 tissue elastances arranged in parallel, the effective elastance can be represented as

$$E_{total} = \left[\sum_{n=1}^{1000}\left(\frac{1}{H_n}\right)\right]^{-1}$$

Given the sufficiently small pressure increment and ample time to equilibration over the PV step, H_n values were assumed equal across all elements, allowing the relationship between recruitment and elastance to be approximated as

$$E_{total} = \frac{H_{tissue}}{N_{open}} = \frac{H_{tissue}}{N_{total}\ x\ f_{open}}.$$

The ratio $\frac{H_{tissue}}{N_{total}}$ can be estimated from the mechanical data as the best estimate of elastance of the fully recruited lung – the minimum PV step elastance at a PEEP of 6 cm H_2O. This metric was chosen in order to minimize contributions of recruitment at low levels of PEEP and strain stiffening at high PEEP. The fraction of collapsed acini can then be modeled as

$$p_{collapse}(PEEP) = \%_{obstructed} + \left(1 - \frac{min\{E_{step}(6cmH_2O)\}}{H(PEEP)}\right)$$

where $\%_{obstructed}$ is the fraction of tissue occluded by cells/debris on histology, $H(PEEP)$ is the constant phase elastance estimated at a given PEEP and $min\{E_{step}(6cmH_2O)\}$ is the estimate of intrinsic stiffness of the fully recruited lung described above. A uniformly distributed random number between 0 and 1 is drawn each time a terminal tissue element is reached – if this number is greater than $p_{collapse}$, acinar mechanics are computed from the distribution according to the appropriate tissue mechanical model above, however, if this number is less than $p_{collapse}$ then the terminal element's elastance value is made equal to 10^{16}, effectively rendering it unable to be ventilated.

For each experimental condition, a posterior distribution of tissue elastances was generated from the 8 proposed models and the observed data. The resulting distributions were stable to repeated simulation. Cumulative distributions generated under each model vary in their exact prediction, however tendency for a leftward shift (toward decreased elastance) is consistent for the 80-week-old Sftpd(-/-) mouse across all models. Models that exclusively use RAC predict minimal age-related change, or even a slight increase, in elastance for the 80-week-old C57Bl6/J mouse. When only elastin fiber thickness is incorporated, the 80-week-old C57Bl6/J demonstrates a dramatic shift in its predicted tissue elastance distribution (greater than that in the Sftpd(-/-) mouse when this is the only factor). When both RAC and fiber thickness are incorporated into the simulation, the 80-week-old Sftpd(-/-) and C57Bl6/J elastance distributions appear similar. In all models tested the 8-week-old Sftpd(-/-) mouse has a left shifted elastance

distribution, which is not evident at 27 weeks. This may reflect a sampling bias, as the number of walls without apparent fibers increases with age and loss of Sftpd, but was not incorporated into the model. As fibers below a certain thickness often fail to retain dye during the contrast enhancing steps of the procedure, this provides a significant limitation in reliably simulating fiber thickness. Summary statistics on elastance fiber distributions are provided below.

		Fiber thickness (um)		
		Mean	Stdev	CV
WT	8	0.385	0.147	38.2
	27	0.415	0.192	46.3
	80	0.263	0.094	35.7
Sftpd-/-	8	0.344	0.16	46.5
	27	0.399	0.183	45.9
	80	0.297	0.132	44.4
