# Supplementary figures and images for: Histologic and biochemical alterations predict pulmonary mechanical dysfunction in aging mice with chronic lung inflammation
Source: PLoS Comput Biol. 2017 Aug 24;13(8):e1005570. doi: 10.1371/journal.pcbi.1005570 (PMC5570219; doi:10.1371/journal.pcbi.1005570)

**Figure S1: Experimental  $Z_{RS}$  Spectra**

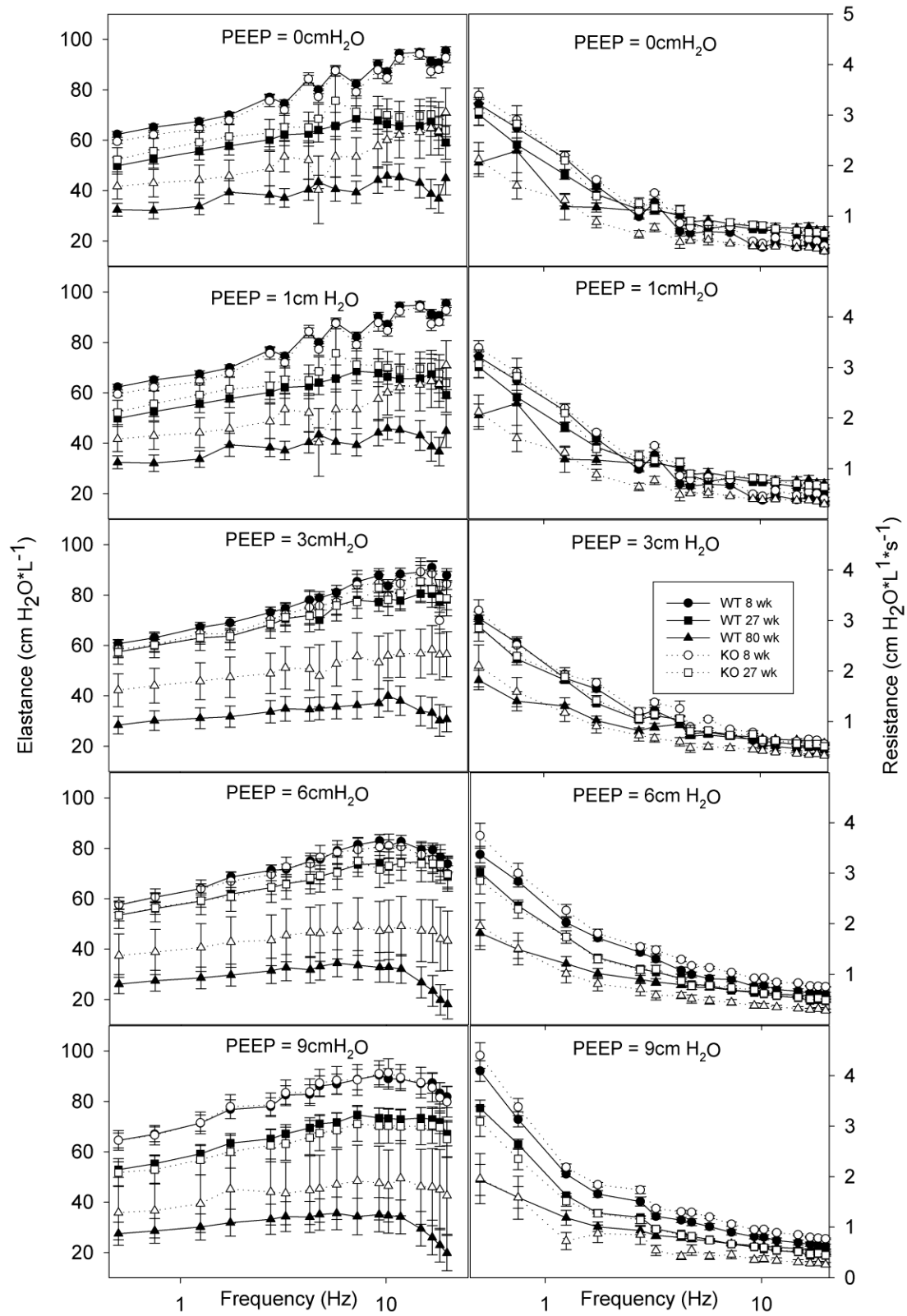

Supplement: S1 Fig — (PDF) [file pcbi.1005570.s002.pdf]

Figure S2: Estimated Constant Phase Parameters

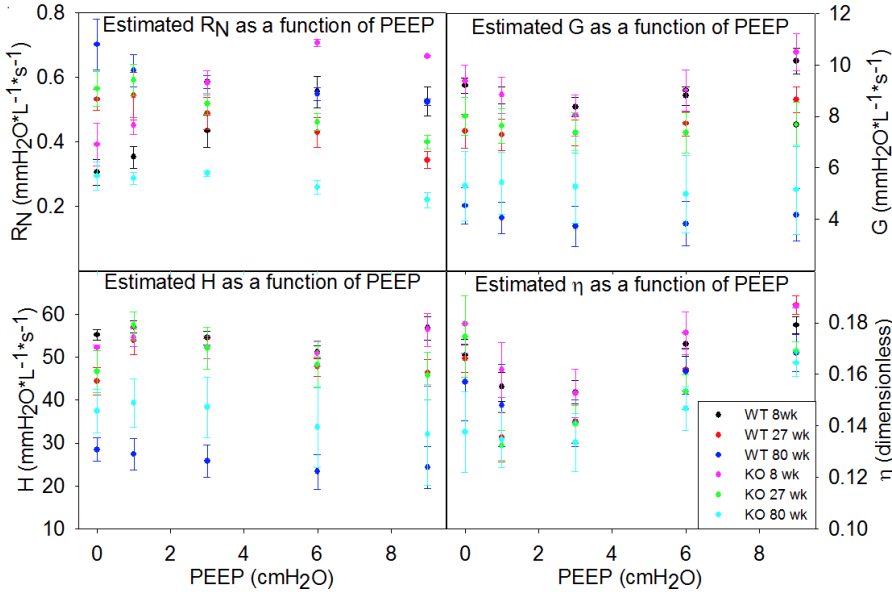

Supplement: S2 Fig — (PDF) [file pcbi.1005570.s003.pdf]

**Figure S3: Residual Model Errors with and without Differential Recruitment**

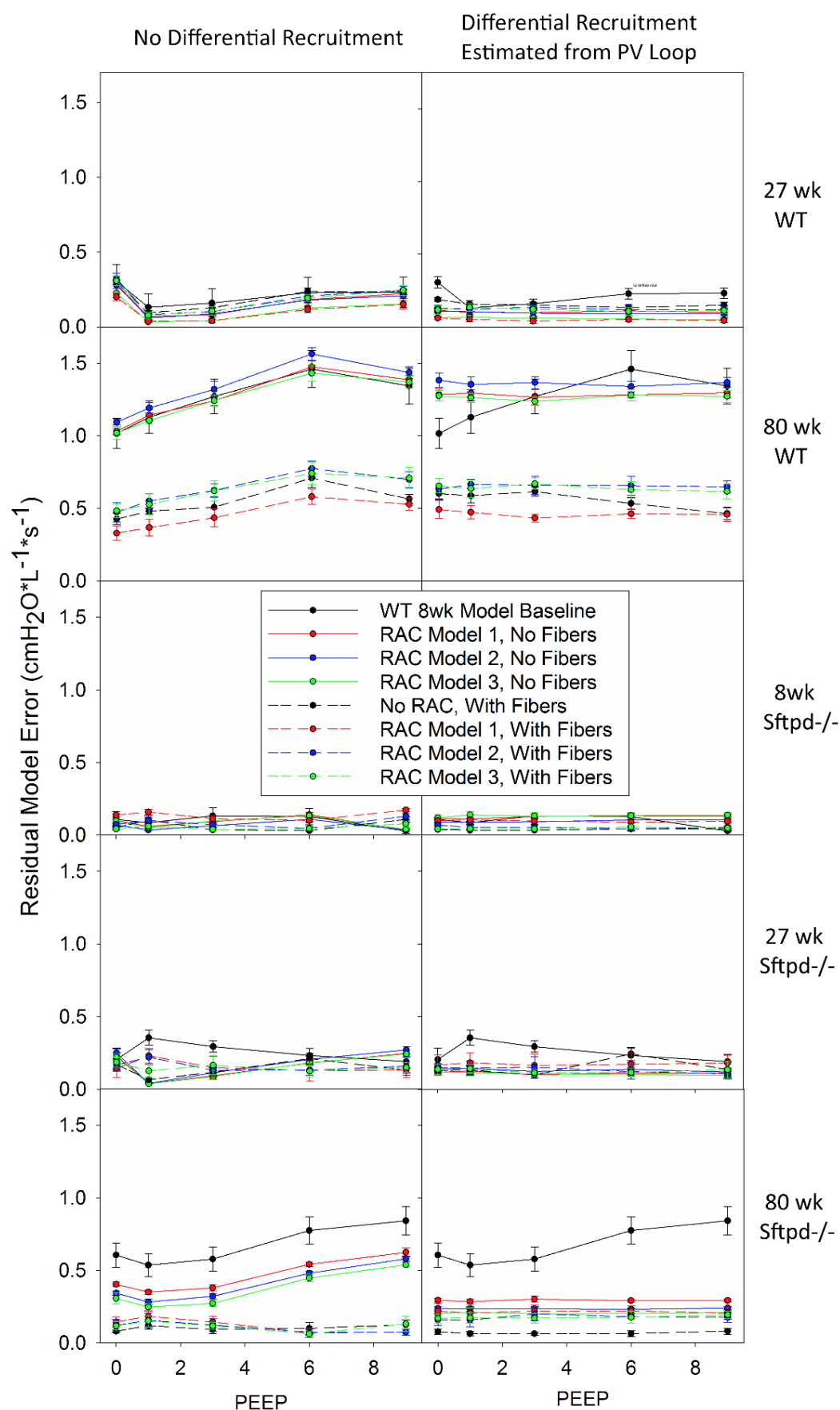

Supplement: S3 Fig — (PDF) [file pcbi.1005570.s004.pdf]
