# Supplementary material for: Histologic and biochemical alterations predict pulmonary mechanical dysfunction in aging mice with chronic lung inflammation
Source: PLoS Comput Biol. 2017 Aug 24;13(8):e1005570. doi: 10.1371/journal.pcbi.1005570 (PMC5570219; doi:10.1371/journal.pcbi.1005570)

Figure S4: Tissue elastance distributions under each model for each experimental group: Histograms of each simulation's tissue elastance distribution are shown as blue bars, with the red curve representing the distribution of the 8wk WT control. Top set of 24 panels use no fiber thickness, while the bottom panels incorporate fiber thickness. Aging results in a substantial left shift in the elastance distribution that is exacerbated by loss of Sftpd under all model assumptions.

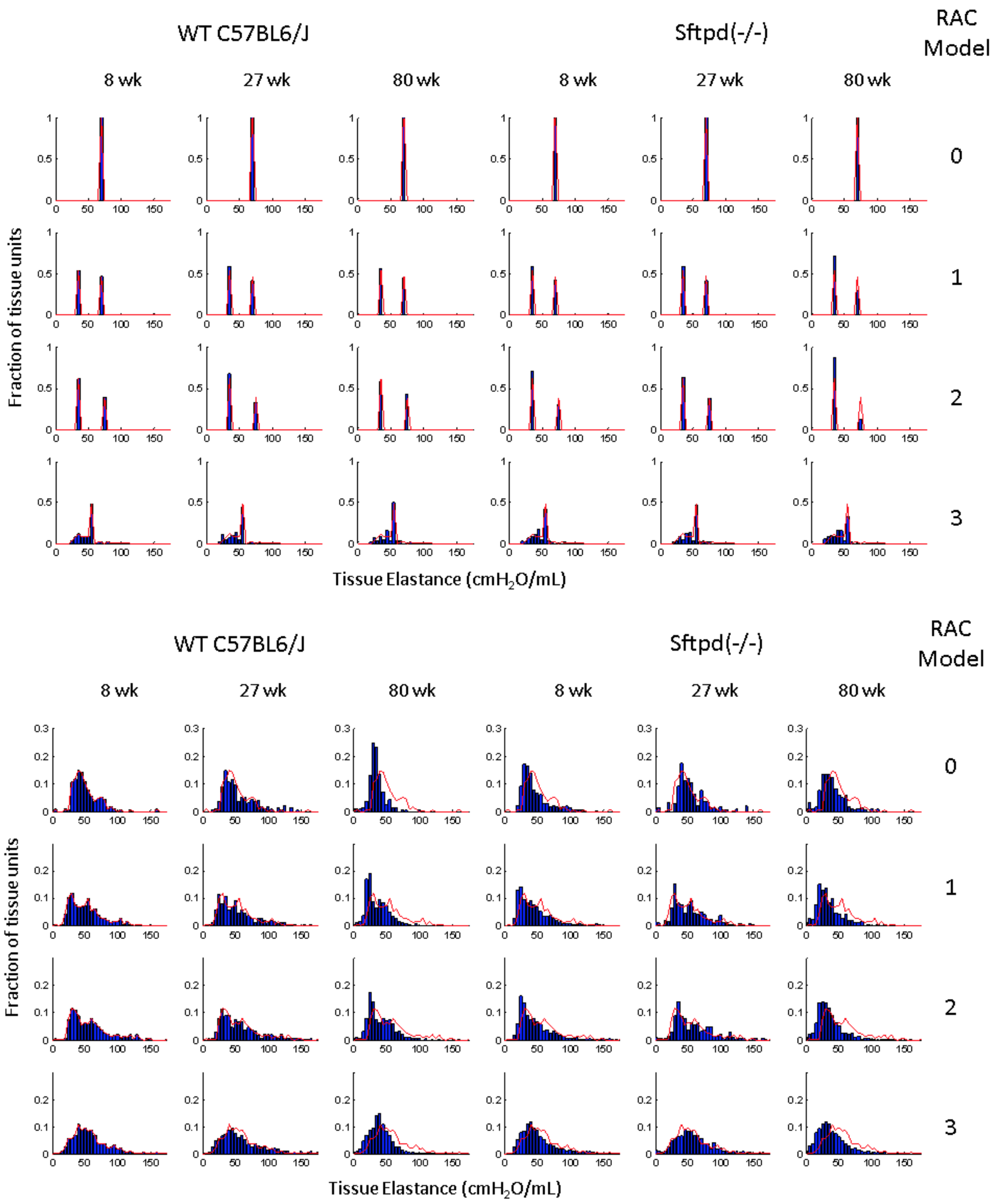

Supplement: S4 Fig — (PDF) [file pcbi.1005570.s005.pdf]
