# Supplementary material for: Histologic and biochemical alterations predict pulmonary mechanical dysfunction in aging mice with chronic lung inflammation
Source: PLoS Comput Biol. 2017 Aug 24;13(8):e1005570. doi: 10.1371/journal.pcbi.1005570 (PMC5570219; doi:10.1371/journal.pcbi.1005570)
Supplement: S5 Fig — (PDF) [file pcbi.1005570.s006.pdf]

Figure S5: Representative spectra of the best fit model shown with experimental data. Experimental (black) and simulated (red) spectra for each experimental condition are shown. Simulated spectra from the model with the maximum likelihood are shown as mean +/- 95% confidence interval determined from repeated simulation. Experimental data are mean +/- standard error.

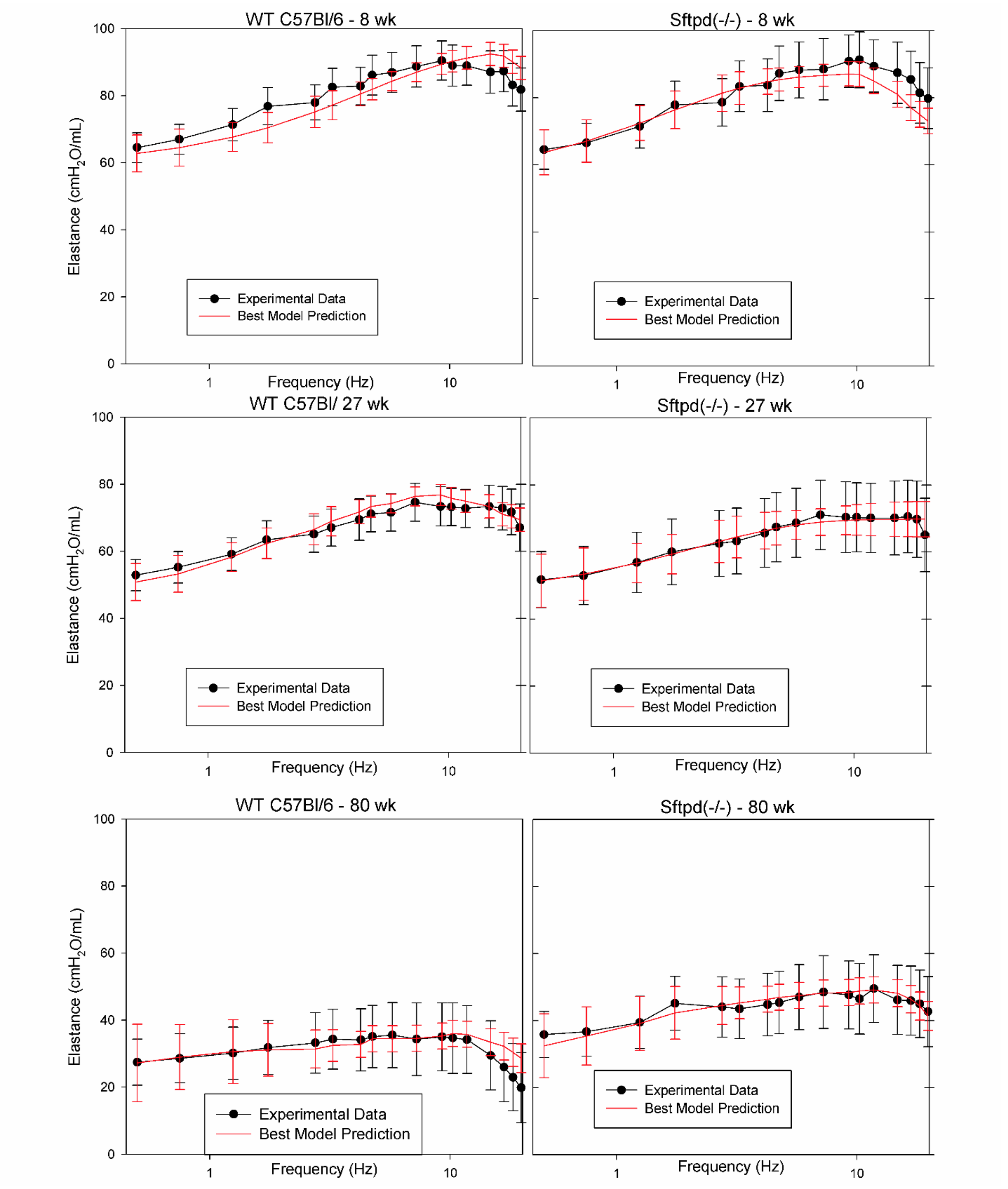

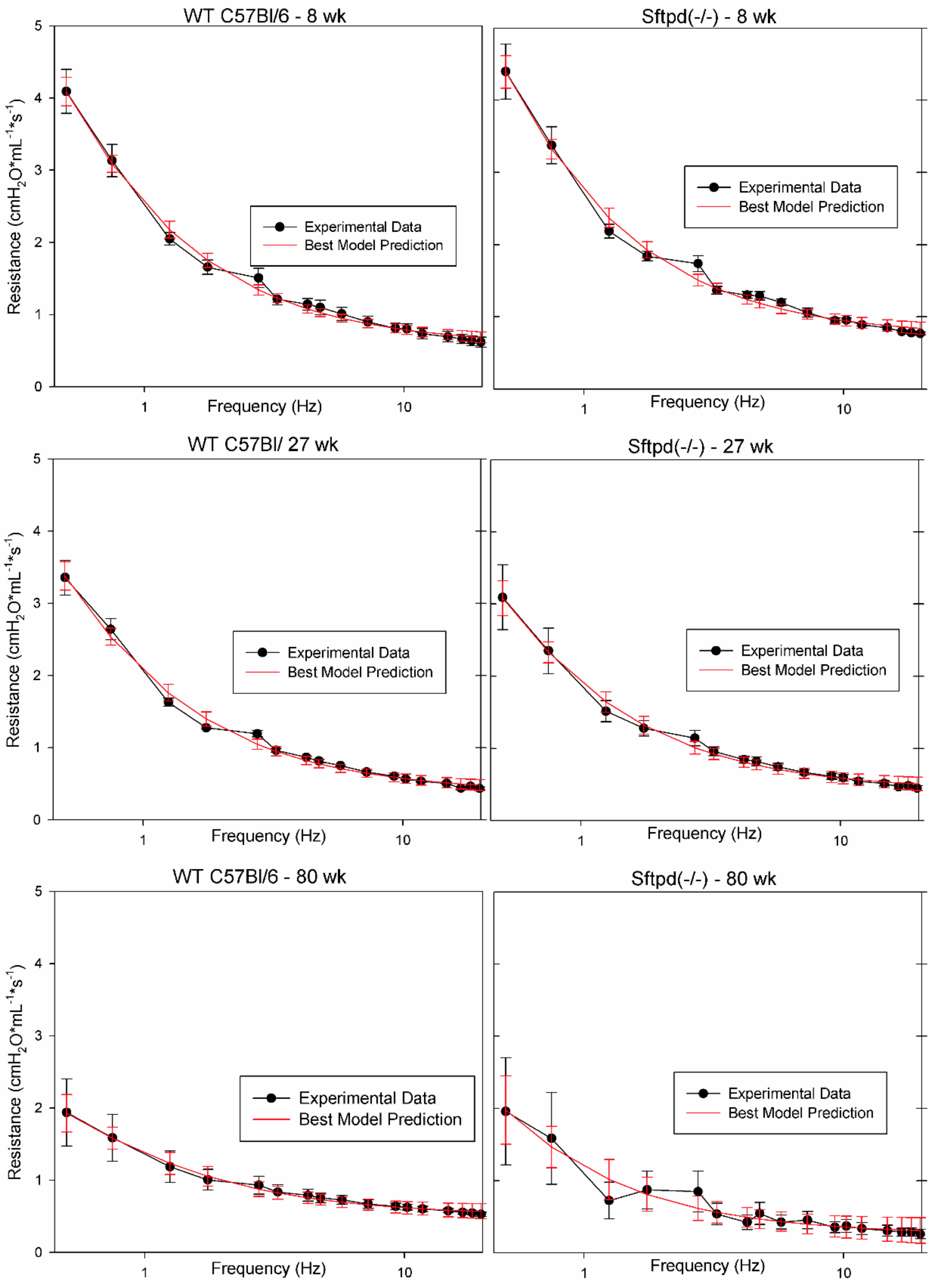

WT C57Bl/6 - 8 wk
Sftpd(-/-) - 8 wk
WT C57Bl/ 27 wk
Sftpd(-/-) - 27 wk
WT C57Bl/6 - 80 wk
Sftpd(-/-) - 80 wk
Resistance (cmH2O*mL-1*s-1)
Frequency (Hz)
Experimental Data
Best Model Prediction
0
1
2
3
4
5
1
10
