# Supplementary material for: Histologic and biochemical alterations predict pulmonary mechanical dysfunction in aging mice with chronic lung inflammation
Source: PLoS Comput Biol. 2017 Aug 24;13(8):e1005570. doi: 10.1371/journal.pcbi.1005570 (PMC5570219; doi:10.1371/journal.pcbi.1005570)
Supplement: S1 Table — (PDF) [file pcbi.1005570.s007.pdf]

Table S1: PEEP dependent error variance

		0	1	2	3	0	1	2	3
		-	-	-	-	+	+	+	+
WT	8	1.1%	4.5%	3.0%	5.9%	12.0%	96.9%	4.4%	4.2%
	27	0.3%	0.3%	0.3%	0.3%	3.9%	0.4%	1.0%	0.6%
	80	22.5%	25.3%	19.6%	23.4%	22.5%	39.5%	22.4%	15.5%
KO	8	1.0%	6.5%	5.6%	8.7%	10.4%	11.5%	75.7%	5.1%
	27	1.3%	1.2%	0.8%	0.8%	29.4%	37.0%	29.3%	180.0%
	80	3.4%	4.6%	4.3%	6.0%	45.9%	4.0%	7.9%	1.5%

Reduction in the variance of the model error across PEEP levels following incorporation of stochastic collapse. The variance in error with PEEP was calculated for each model before and following the addition of PEEP-dependent derecruitment. The ratio of these data represents the percent of the variance in error with PEEP remaining after the addition of recruitment based on PV-loop behavior. In general, the addition of derecruitment dramatically reduced the PEEP-dependence of the error term, independent of model assumption. Error as a function of PEEP is shown for each model and condition below.

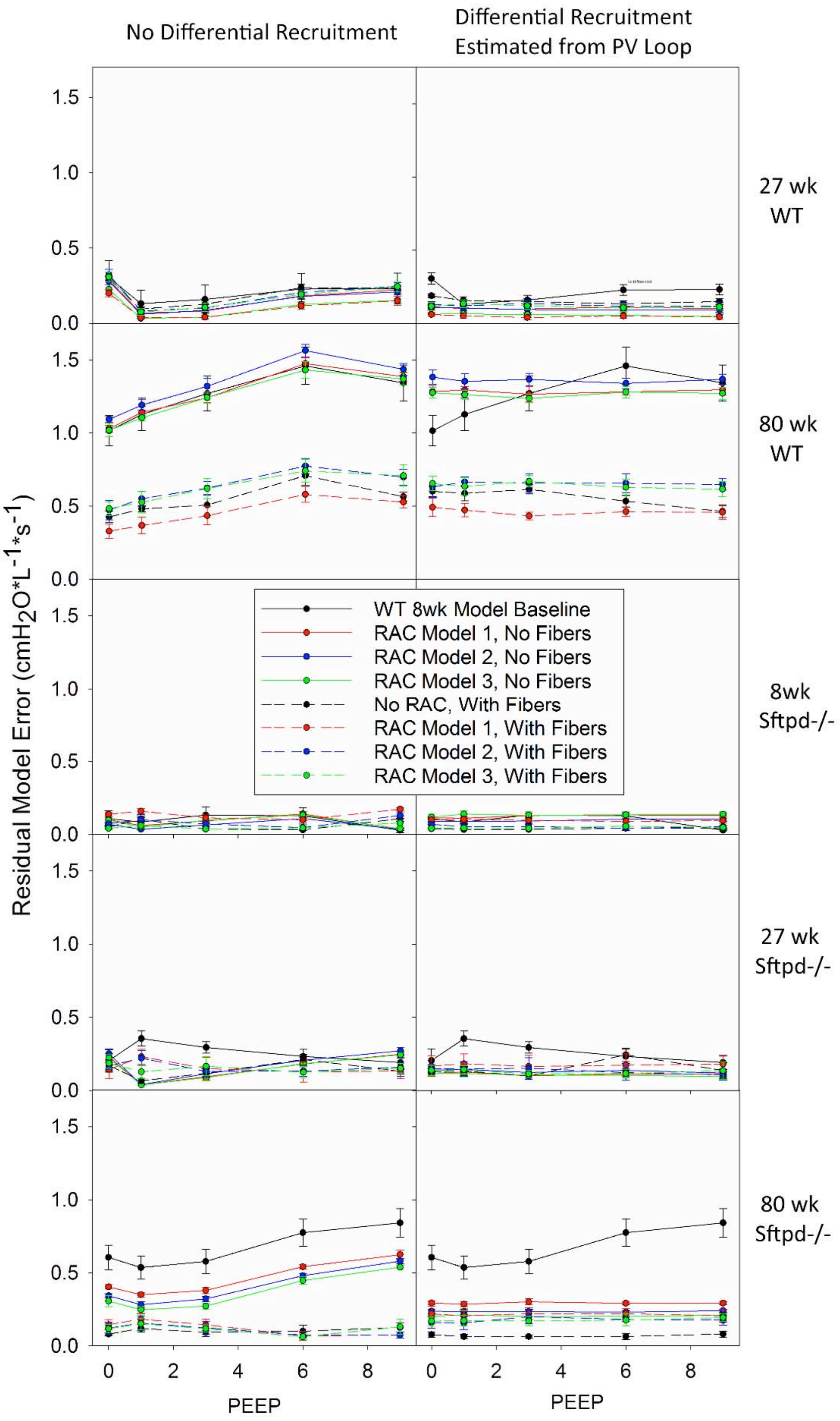
No Differential Recruitment
Differential Recruitment Estimated from PV Loop
27 wk WT
80 wk WT
8wk Sftpd-/-
27 wk Sftpd-/-
80 wk Sftpd-/-
Residual Model Error (cmH2O*L-1*s-1)
PEEP
WT 8wk Model Baseline
RAC Model 1, No Fibers
RAC Model 2, No Fibers
RAC Model 3, No Fibers
No RAC, With Fibers
RAC Model 1, With Fibers
RAC Model 2, With Fibers
RAC Model 3, With Fibers
